# First-Line Treatments for Extensive-Stage Small-Cell Lung Cancer With Immune Checkpoint Inhibitors Plus Chemotherapy: A Network Meta-Analysis and Cost-Effectiveness Analysis

**DOI:** 10.3389/fonc.2021.740091

**Published:** 2022-01-19

**Authors:** Shuo Kang, Xinchen Wang, Yue Zhang, Boyuan Zhang, Fangjian Shang, Wei Guo

**Affiliations:** ^1^ School of Pharmacy, Hebei Medical University, Shijiazhuang, China; ^2^ Department of Pathology, Handan Central Hospital, Handan, China; ^3^ Department of Immunology, Hebei Medical University, Shijiazhuang, China; ^4^ School of Public Health, Hebei Medical University, Shijiazhuang, China; ^5^ Department of General Surgery, The First Hospital of Hebei Medical University, Shijiazhuang, China

**Keywords:** immune checkpoint inhibitors, chemotherapy, extensive-stage small-cell lung cancer, cost-effectiveness, first-line treatment

## Abstract

**Background:**

Immune checkpoint inhibitors (ICIs) plus chemotherapy were unlikely to be considered cost-effective compared with chemotherapy as the first-line treatment of patients with extensive-stage small-cell lung cancer (ES-SCLC) in China due to its high costs. However, the cost-effectiveness of the comparison between the regimens of ICIs plus chemotherapy were remained unclear yet. The aim of this study was to evaluate the efficacy and cost-effectiveness of ICIs plus chemotherapy as the first-line treatment for ES-SCLC from the perspective of the Chinese healthcare system.

**Methods:**

A network meta-analysis (NMA) was conducted to indirect compare the clinical benefits between the ICIs plus chemotherapy regimens. A decision-analytic model was established to evaluate the cost-effectiveness from the Chinese healthcare system, the clinical efficacy and safety data were obtained from the clinical trials and the results of NMA. Cost and utility values were gathered from the local charges and previously studies. Key outputs of the NMA were overall survival (OS) and progression-free survival (PFS). Incremental cost-effectiveness ratios (ICERs) were estimated. One-way and probabilistic sensitivity analyses were performed to explore the robustness of the model outcomes.

**Results:**

Five clinical trials (IMpower133, CASPIAN, KEYNOTE-604, CA184-156, and EA5161) of 1,255 patients received first-line ICIs plus chemotherapy strategies were analyzed in the NMA. NMA showed that nivolumab plus chemotherapy was ranked higher than other strategies. The cost-effectiveness analysis showed that atezolizumab plus chemotherapy achieved relatively higher health benefits and lower costs. One-way sensitivity analyses revealed that the cost of ICIs had the substantial impact on model outcomes. The probabilistic sensitivity analyses suggested that the probability of atezolizumab plus chemotherapy could be considered cost-effective was more than 50% at the willingness-to-pay (WTP) threshold of $31,313/QALY in China. In scenario analyses, when the price of nivolumab reduced 80%, the probability of nivolumab plus chemotherapy being cost-effective was more than 50%.

**Conclusions:**

The NMA and cost-effectiveness revealed that atezolizumab plus chemotherapy is the most favorable first-line treatment for previously untreated ES-SCLC patients compared other ICIs plus chemotherapy regimens in China. The price reduction of nivolumab would make nivolumab plus chemotherapy be the most cost-effective option in future possible context.

## Introduction

The Global Burden of Disease Study revealed that lung cancer is one of the leading causes of non-communicable disease burden worldwide (1). Nearly 13–17% of all lung cancers are small-cell lung cancer (SCLC), which is characterized by rapid doubling time and showed the propensity for early development of widespread metastases. The latest epidemiological survey presented that the survival of SCLC remained low and stayed at 14–15% (2). SCLC is the most aggressive type of all lung cancers and with the inferior prognosis (3, 4). Approximately two-thirds of all SCLC are progressed to extensive-stage (ES) at the time of initial diagnosis (3), which with a two-year survival rate is less than 5% by treating with the standard first-line platinum-doublet chemotherapy strategy (4, 5). Therefore, the development of novel drugs to manage ES-SCLC is necessary (6–8).

Immune checkpoint inhibitors (ICIs), could reduce the immunosuppression in the tumor microenvironment and reactivate the antitumor function of T cells through inhibiting the cytotoxic T lymphocyte-associated protein 4 (CTLA-4) and PD-1/PD-L1 pathway (programmed cell death-1 and programmed cell death receptor ligand-1 pathway) (9–13). Although ipilimumab plus chemotherapy did not show the significant clinical benefit in CA184-156 trial in the initial exploration, subsequent IMpower133, CASPIAN, KEYNOTE-604, and EA5161 trial revealed that atezolizumab plus chemotherapy, durvalumab plus chemotherapy, pembrolizumab plus chemotherapy, and nivolumab plus chemotherapy could be considered potential first-line treatments for patients with ES-SCLC because these strategies could significantly reduce the risk of disease progression and death in comparison with standard platinum-based first-line chemotherapy (14–18). ICIs has changed the traditional treatment paradigm for previously untreated ES-SCLC with the approval of PD-L1 inhibitors atezolizumab and durvalumab plus chemotherapy in China and United states, though the two above PD-1 inhibitors plus chemotherapy have not been approved for ES-SCLC in China (19, 20). Although previous studies have shown that ICIs plus chemotherapy regimens are unlikely to be cost-effective in the first-line treatment for ES-SCLC when compared with chemotherapy in China due to the high price of ICIs (21, 22), determining the most cost-effective regimen and clearing the first treatment option among above five ICIs plus chemotherapy strategies was also meaningful and helpful for the clinical oncologists and Chinese healthcare decision makers. However, no relevant study has directly compared the above five ICIs plus chemotherapy regimens with each other, so we conducted a network meta-analysis. The objective of our study was to evaluate the cost-effectiveness of atezolizumab plus chemotherapy, durvalumab plus chemotherapy, pembrolizumab plus chemotherapy, nivolumab plus chemotherapy, and ipilimumab plus chemotherapy as first-line treatments for previously untreated ES-SCLC from the perspective of the Chinese healthcare system.

## Methods

### Network Meta-Analysis

#### Study Selection and Assessment of the Risk of Bias

The network meta-analysis was followed the PRISMA guidelines. The electronic databases, namely, PubMed, EMBASE and the Cochrane Central Register of Controlled Trials were searched to identify the eligible randomized controlled trials (RCTs) that compared PD-1/PD-L1 or CTLA-4 inhibitors plus chemotherapy with chemotherapy for patients with previously untreated ES-SCLC, eligible studies with the deadline up to June 22, 2021. We also searched the meeting abstracts presented at the European Society of Medical Oncology (ESMO), the American Society of Clinical Oncology (ASCO), the American Association for Cancer Research (AACR), and the World Conference on Lung Cancer (WCLC). Owing to the absence of the price information, ICIs that were not approved in China were not considered in our analysis such as avelumab. Publications which were not written in English were ineligible, and only the latest data of the same trial were considered for the network meta-analysis. The risk of bias of clinical trials were evaluated in RevMan software (version 5.3) based on Cochrane Collaboration’s tool (23).

#### Collection of Data

Independently screened studies were conducted by two reviewers (SK and XCW). The collected data included study characteristics, treatment strategies, and the HRs (hazard ratios) for PFS and OS.

#### Synthesis of Data and Statistical Analysis

We performed the network meta-analysis to obtain the HRs of PFS and OS between the regimens of ICIs plus chemotherapy based on the Bayesian methods. A fixed-effects model was used for the analysis due to the absence of data to assess the heterogeneity between trials, and the consistency test was exempted because of the deficiency of a closed loop for the indirect comparison (24, 25), ranking the different strategies in terms of the surface under the cumulative ranking curve (SUCRA). Statistical analysis was conducted by using R software (version 4.0.5) with “gemtc” package.

### Cost-Effectiveness Analysis

#### Analytical Overview and Model Structure

**Figure 1:**
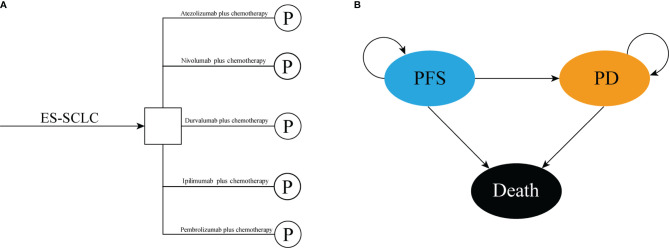
The structure of the **(A)** decision tree and **(B)** partitioned survival model. ES-SCLC, extensive-stage small-cell lung cancer; PFS, progression-free survival; PD, progressed disease.

We conducted a mathematical model that combined decision tree and partitioned survival model to assess the cost-effectiveness of the following five competing regimens for previously untreated extensive-stage small-cell lung cancer, that included atezolizumab plus chemotherapy, durvalumab plus chemotherapy, pembrolizumab plus chemotherapy, nivolumab plus chemotherapy, and ipilimumab plus chemotherapy (**Figure 1A**). The partitioned survival model included the following three mutually exclusive health states reflecting different characteristics of the disease: progression-free survival (PFS), progressed disease (PD), and death (**Figure 1B**). The cycle length of the partitioned survival model was set to be three weeks, and the time horizon was ten years, the initial health state of all the patients was PFS, and the patients either remained in their assigned health state or redistributed to another health state during each cycle. The proportion of patients in each state of each cycle was determined by the PFS rates and OS rates which were obtained from the clinical trials and the NMA. The following hypothetical patient cohort demographics when entering the partitioned survival model matched those of the patients in the IMpower133, CASPIAN, KEYNOTE-604, and CA184-156 trials: 64 years old and histologically or cytologically confirmed ES-SCLC with not previously systemic therapy treated. The main outcomes included total costs, life-years (LYs), quality-adjusted life years (QALYs), and incremental cost-effectiveness ratios (ICERs) were estimated. We also estimated the incremental net-monetary benefits (INMBs) based on the following formula: INMB = (E_1_ − E_2_) ∗ λ − (C_1_ − C_2_) = ΔE ∗ λ − ΔC, where λ was the willingness-to-pay (WTP) threshold in China. Costs and QALYs were discounted at an annual rate of 5% according to Chinese guidelines for pharmacoeconomic evaluations (26). All costs were shown in 2020 US dollars (US $1 = CNY ¥6.898). Three times of the per capita gross domestic product (GDP) of China in 2020 (US $31,313/QALY) was used as the WTP threshold to judge the cost-effectiveness of the five competing strategies (27).

#### Clinical Data

Clinical efficacy and safety data were obtained from the IMpower133, CASPIAN, KEYNOTE-604, CA184-156, and EA5161 trials. In clinical trials, Atezolizumab plus chemotherapy, durvalumab plus chemotherapy, pembrolizumab plus chemotherapy, ipilimumab plus chemotherapy, and nivolumab plus chemotherapy could make the median progression-free survival reach 5.2 months (95%CI: 4.4–5.6), 5.1 months (95%CI: 4.7–6.2), 4.5 months (95%CI: 4.3–5.4), 4.6 months (95%CI: 4.5–5.0), and 5.5 months, respectively. The five first-line regimens could extent the median overall survival to 12.3 months (95%CI: 10.8–15.9), 12.9 months (95%CI: 11.3–14.7), 10.8 months (95%CI: 9.2–12.9), 11.0 months (95%CI: 10.5–11.3), and 11.3 months, respectively. We reconstructed the individual patient time-to-event data and extrapolated the Kaplan–Meier (KM) curves beyond the follow-up duration of the clinical trials by fitting the parametric survival functions among: exponential, gamma, Weibull, log-normal, log-logistic, and Gompertz (28). The GetData Graph Digitizer software (version 2.26; http://www.getdata-graphdigitizer.com/index.php) was used to extract the data point from the KM curves, and R software (version 4.0.5) was used for statistical analysis. Visual inspection and Akaike information criterion (AIC) were used to judge the goodness of model fitting. AIC values and best fitted models are shown in **Supplementary Table 1**. The comparison between fitting curves and KM curves of the four ICIs plus chemotherapy regimens are shown in **Supplementary Figures 1–4**. The above algorithm was not applicable to nivolumab plus chemotherapy due to the incomplete data disclosed in EA5161 trial. Similar to the previously study done, we used the survival data of durvalumab plus chemotherapy as the baseline treatment because of the longer mature follow-up time (29). The PFS and OS rates of atezolizumab, pembrolizumab, nivolumab, and ipilimumab plus chemotherapy were estimated by multiplying the survival probabilities of durvalumab plus chemotherapy and HRs of the two treatments against durvalumab plus chemotherapy, respectively, which were obtained from the above NMA. After the disease progressed, the proportion of patients who received second-line treatment was gathered from the clinical trials. However, IMpower133 trial, CASPIAN trial, and CA184-156 trial did not declare the specific subsequent anticancer therapy, thus to simplify the model and reflect patients benefit, we assumed topotecan as the second-line treatment when the disease progressed. In response to the immature data in EA5161 trial, we made reasonable assumptions based on relevant data and examined them in the sensitivity analyses.

#### Cost and Utility Data

This current cost-effectiveness analysis was performed from the perspective of the Chinese healthcare system, so the indirect and hidden costs were not included, only direct medical costs were calculated, namely, drug acquisition costs of first-line and second-line treatments, costs of routine follow-up and best supportive care (BSC), terminal care and management of treatment-related serious adverse events (SAEs, grade ≥3).

Drug administration schedules in the cost-effectiveness analysis were consistent with the clinical trials (14–18). We assumed a typical patient had a body surface area (BSA) of 1.72 m^2^ (height: 1.64 m; weight: 65 kg) to calculate the dose of the agents (30). Currently, atezolizumab patient assistance program (PAP) was conducted for patients with ES-SCLC to improve the drug affordability in China, where the PAP supports patients to pay atezolizumab for the first two cycles, and then they will receive free atezolizumab for the next three cycles, and continue cycling. We considered this PAP in our analysis. The Chinese government adopted the way of national medical insurance negotiation based on the pharmacoeconomic evidence to improve the cost-effectiveness of the high-value innovative drugs. Although the last results of the 2021 national medical insurance negotiation revealed that all of the five ICIs failed to be included in the Nation Medical Insurance List, we also considered the effect of price fluctuations on the robustness of the model results in sensitivity analyses.

Each health state was assigned a utility preference on a scale of 0 (death) to 1 (perfect health). The health state utility values in our model were obtained from the previously published studies, where the utility values of PFS, PD, and death were 0.673, 0.473 and 0, respectively (31, 32). The reported disutility caused by SAEs were also considered in the model. All the key clinical inputs are shown in **Table 1**.

**Table 1:** Model inputs: base-line values, ranges, and distributions for sensitivity analyses.

Parameters	Base-line value	Range		Distribution	Reference
		Minimum	Maximum		
Clinical inputs					
Log-logistic PFS survival model of Durc	Shape = 1.997; Scale = 5.858	ND	ND	Fixed	(16)
Gamma OS survival model of Durc	Shape = 1.463; Rate = 0.086	ND	ND	Fixed	(16)
HR of PFS (Atec versus Durc)	0.96	0.72	1.3	Log-normal	NMA
HR of PFS (Pemc versus Durc)	0.94	0.71	1.2	Log-normal	NMA
HR of PFS (Nivc versus Durc)	0.81	0.55	1.2	Log-normal	NMA
HR of PFS (Ipic versus Durc)	1.1	0.85	1.3	Log-normal	NMA
HR of OS (Atec versus Durc)	0.93	0.67	1.3	Log-normal	NMA
HR of OS (Pemc versus Durc)	1.1	0.8	1.4	Log-normal	NMA
HR of OS (Nivc versus Durc)	0.89	0.58	1.4	Log-normal	NMA
HR of OS (Ipic versus Durc)	1.3	0.96	1.6	Log-normal	NMA
Cost inputs (US $)					
Durvalumab per 1,500 mg	7,866.6	5,899.95	9,833.25	Gamma	Local charge
Atezolizumab per 1,200 mg	4,755	3,566.25	5,943.75	Gamma	Local charge
Pembrolizumab per 200 mg	5,195.1	3,896.33	6,493.88	Gamma	Local charge
Nivolumab per 40 mg	665	498.75	831.25	Gamma	Local charge
Ipilimumab per 1 mg	81.2	60.9	101.5	Gamma	Local charge
Carboplatin per 100 mg	11.7	8.78	14.63	Gamma	Local charge
Cisplatin per 10 mg	2.5	1.88	3.13	Gamma	Local charge
Etoposide per 100 mg	35.5	26.63	44.38	Gamma	Local charge
Topotecan per 1 mg	25	18.75	31.25	Gamma	Local charge
Routine follow-up per cycle	59.2	44.4	74	Gamma	(33)
Best supportive care per cycle	359	169	845	Gamma	(34)
Terminal care	2,176	845	5,812	Gamma	(34)
Neutropenia per event	466	415	508	Gamma	(35)
Anemia per event	537	478	585	Gamma	(35)
Decreased neutrophil count per event	466	0	1,384	Gamma	(36)
Thrombocytopenia per event	6,397	5,117	7,676	Gamma	(37)
Leukopenia per event	466	415	508	Gamma	(35)
Febrile neutropenia per event	953	715	1,191	Gamma	(38)
Diarrhea per event	29	21.75	36.25	Gamma	(39)
Management SAEs in Nivc group	446	255	1,009	Gamma	(40)
Utility inputs					
Utility of PFS	0.673	0.5	0.84	Beta	(31, 32)
Utility of PD	0.473	0.35	0.6	Beta	(31, 32)
Disutility of toxicities					
Neutropenia	−0.2	−0.15	−0.25	Beta	(41)
Anemia	0.073	−0.037	−0.11	Beta	(32)
Decreased neutrophil count	−0.2	−0.15	−0.25	Beta	(41)
Thrombocytopenia	−0.19	−0.143	−0.238	Beta	(41)
Leukopenia	−0.2	−0.15	−0.25	Beta	(41)
Febrile neutropenia	−0.42	−0.315	−0.525	Beta	(41)
Diarrhea	−0.07	−0.0525	−0.0875	Beta	(41)
SAEs in Nivc group	−0.157	−0.118	−0.196	Beta	(10)
Risk of serious adverse events in Atec group					
Neutropenia	0.232	0.173	0.291	Beta	(15)
Anemia	0.141	0.093	0.189	Beta	(15)
Decreased neutrophil count	0.141	0.093	0.189	Beta	(15)
Thrombocytopenia	0.101	0.059	0.143	Beta	(15)
Leukopenia	0.05	0.02	0.08	Beta	(15)
Risk of serious adverse events in Durc group					
Neutrophil	0.24	0.189	0.291	Beta	(16)
Anemia	0.09	0.056	0.124	Beta	(16)
Thrombocytopenia	0.06	0.031	0.089	Beta	(16)
Decreased neutrophil count	0.06	0.031	0.089	Beta	(16)
Febrile neutropenia	0.053	0.026	0.08	Beta	(16)
Risk of serious adverse events in Pemc group					
Neutrophil	0.435	0.37	0.5	Beta	(17)
Anemia	0.157	0.109	0.205	Beta	(17)
Thrombocytopenia	0.139	0.094	0.184	Beta	(17)
Leukopenia	0.117	0.075	0.159	Beta	(17)
Risk of serious adverse events in Ipic group					
Diarrhea	0.07	0.047	0.093	Beta	(14)
Neutropenia	0.14	0.109	0.171	Beta	(14)
Anemia	0.08	0.056	0.104	Beta	(14)
Decreased neutrophil count	0.07	0.047	0.093	Beta	(14)
Risk of serious adverse events in Nivc group					
SAEs	0.77	0.578	0.963	Beta	(18)
Others					
Proportion of patients received carboplatin in Durc group	0.75	0.698	0.802	Beta	(16)
Proportion of patients received carboplatin in Pemc group	0.75	0.689	0.802	Beta	(17)
Proportion of patients received carboplatin in Ipic group	0.65	0.611	0.689	Beta	(14)
Proportion of patients received carboplatin in Nivc group	0.6	0.45	0.75	Beta	assumed
Proportion of patients received second-line treatment in Atec group	0.502	0.433	0.571	Beta	(15)
Proportion of patients received second-line treatment in Durc group	0.44	0.381	0.499	Beta	(16)
Proportion of patients received second-line treatment in Pemc group	0.53	0.464	0.596	Beta	(17)
Proportion of patients received second-line treatment in Ipic group	0.48	0.435	0.525	Beta	(14)
Proportion of patients received second-line treatment in Nivc group	0.5	0.375	0.625	Beta	assumed
Discount rate	0.05	0	0.08	ND	(26)

PFS, progression-free survival; OS, overall survival; ND, not determined; HR, hazard ratio; NMA, network meta-analysis; Durc, durvalumab plus chemotherapy; Atec, atezolizumab plus chemotherapy; Pemc, pembrolizumab plus chemotherapy; Nivc, nivolumab plus chemotherapy; Ipic, ipilimumab plus chemotherapy; SAEs, serious adverse events; PD, progressed disease.

#### Sensitivity Analyses

One-way and probabilistic sensitivity analyses (PSA) were conducted to test the robustness of the model outcomes. In the one-way sensitivity analyses, parameters were changed one-by-one over its preset plausible range to estimate which parameter plays a vital role on the model outcomes. The plausible range of each parameter was based on the 95% confidence intervals obtained from the published studies or by assuming ±25% of the base-case values when the data were not available. The plausible ranges are shown in **Table 1**. The results of one-way sensitivity analyses were performed in the Tornado diagram. For the PSA, a Monte Carlo simulation of 1,000 replications was conducted by jointly sampling the key model parameters from the pre-specified statistical distribution. Log-normal distribution was selected for HRs between the competing regimens, gamma distribution for costs, and beta distribution for proportions, incidence rates, and utility values (42). That was, a set of 1,000 estimated outcomes was gathered, and this data set was used to create the cost-effectiveness acceptability curves (CEACs), which represented the probability of each competing strategy that would be considered cost-effective at various WTP thresholds.

## Results

### Network Meta-Analysis

After searching databases and selecting literatures, a total of five clinical trials (IMpower133, CASPIAN, KEYNOTE-604, CA184-156, and EA5161) involving 1,255 patients were included in the network meta-analysis (**Supplementary Figure 5**) from the identified 19,273 records. The risk of bias judgements for the included studies is presented in **Supplementary Figure 7**. Nivolumab plus chemotherapy was ranked higher than other compared strategies based on the HRs for PFS and OS **(** **Supplementary Figure 8** **).** The HRs for PFS and OS of the indirect comparisons between five regimens are shown in **Table 2**.

**Table 2:** Hazard ratios (green cell) of the network meta-analysis of the progression-free survival and overall survival.

Progression-free survival					
	Atec	Durc	Pemc	Nivc	Ipic
Atec	1	0.96 (95%CI: 0.72–1.3)	1.0 (95%CI: 0.76–1.4)	1.2 (95%CI: 0.79–1.8)	0.91 (95%CI: 0.7–1.2)
Durc	1.0 (95%CI: 0.78–1.4)	1	1.1 (95%CI: 0.81–1.4)	1.2 (95%CI: 0.83–1.8)	0.94 (95%CI: 0.75–1.2)
Pemc	0.97 (95%CI: 0.72–1.3)	0.94 (95%CI: 0.71–1.2)	1	1.2 (95%CI: 0.78–1.7)	0.88 (95%CI: 0.7–1.1)
Nivc	0.84 (95%CI: 0.56–1.3)	0.81 (95%CI: 0.55–1.2)	0.87 (95%CI: 0.58–1.3)	1	0.77 (95%CI: 0.53–1.1)
Ipic	1.1 (95%CI: 0.86–1.4)	1.1 (95%CI: 0.85–1.3)	1.1 (95%CI: 0.89–1.4)	1.3 (95%CI: 0.91–1.9)	1
**Overall survival**					
	Atec	Durc	Pemc	Nivc	Ipic
Atec	1	0.93 (95%CI: 0.67–1.3)	0.87 (95%CI: 0.62–1.2)	1.0 (95%CI: 0.66–1.7)	0.74 (95%CI: 0.55–1.0)
Durc	1.1 (95%CI: 0.77–1.5)	1	0.94 (95%CI: 0.71–1.3)	1.1 (95%CI: 0.73–1.7)	0.8 (95%CI: 0.63–1.0)
Pemc	1.1 (95%CI: 0.81–1.6)	1.1 (95%CI: 0.8–1.4)	1	1.2 (95%CI: 0.77–1.8)	0.85 (95%CI: 0.66–1.1)
Nivc	0.96 (95%CI: 0.6–1.5)	0.89 (95%CI: 0.58–1.4)	0.84 (95%CI: 0.54–1.3)	1	0.71 (95%CI: 0.47–1.1)
Ipic	1.3 (95%CI: 0.99–1.8)	1.3 (95%CI: 0.98–1.6)	1.2 (95%CI: 0.91–1.5)	1.4 (95%CI: 0.94–2.1)	1

Atec, atezolizumab plus chemotherapy; Durc, durvalumab plus chemotherapy; Pemc, pembrolizumab plus chemotherapy; Nivc, nivolumab plus chemotherapy; Ipic, ipilimumab plus chemotherapy.

### Cost-Effectiveness Analysis

#### Base-Case Analysis

From the perspective of the Chinese healthcare system, in a ten-year horizon, nivolumab plus chemotherapy could bring the greatest clinical benefit followed by atezolizumab plus chemotherapy; the cost of atezolizumab plus chemotherapy is the lowest among the five competing regimens due to the PAP. The cost of ipilimumab plus chemotherapy is the most and the QALYs obtained was least, and it suggested that ipilimumab plus chemotherapy is the dominated strategy. The base-case results revealed that atezolizumab plus chemotherapy could be considered the most cost-effective option for previously untreated ES-SCLC. The health benefit and economic outcomes are summarized in **Table 3**.

**Table 3:** Base-case results.

Strategy	Total Cost, $	LYs	QALYs	ICER ($/QALY, versus Pemc)	INMB (versus Pemc)
Pemc	72,012.27	1.34	0.75	–	–
Durc	90,750.92	1.45	0.79	469,482.10	-17,488.84
Atec	41,194.22	1.54	0.83	Dominate	33,381.86
Nivc	87,897.01	1.60	0.88	119234.60	-11,713.14
Ipic	249,215,23	1.18	0.66	Dominated	-179,930.64

LYs, life-years; QALYs, quality-adjusted life years; ICER, incremental cost-effectiveness ratio; INMB, incremental net-monetary benefit; Pemc, pembrolizumab plus chemotherapy; Durc, durvalumab plus chemotherapy; Atec, atezolizumab plus chemotherapy; Nivc, nivolumab plus chemotherapy; Ipic, ipilimumab plus chemotherapy.

#### Sensitivity Analyses

**Figure 2:**
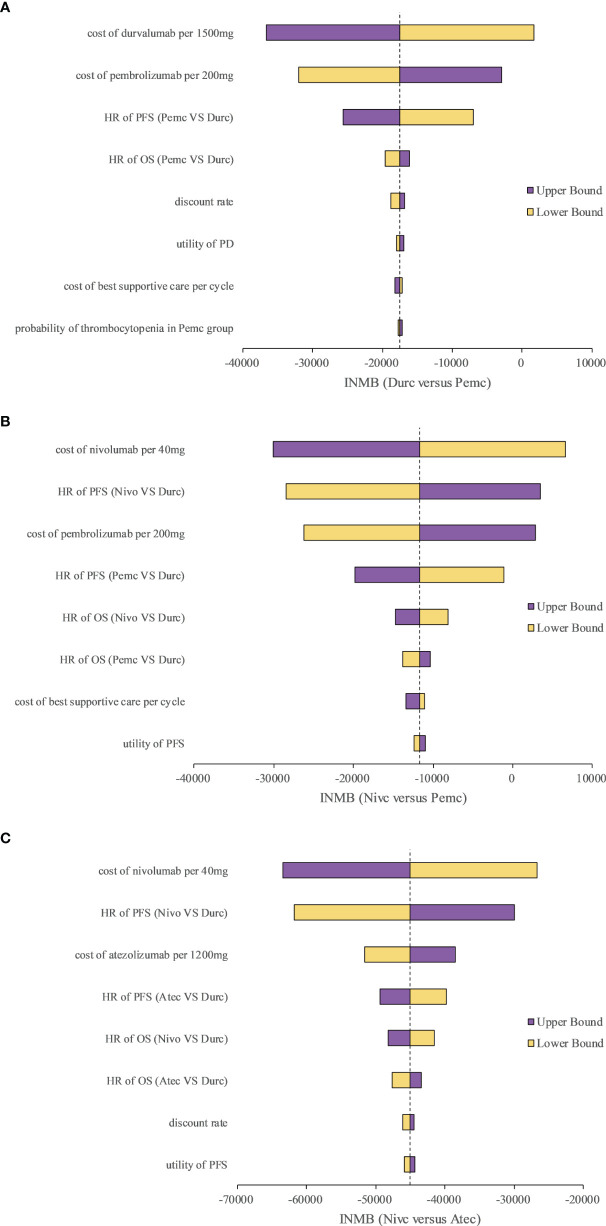
Tornado diagram of one-way sensitivity analyses with greatest influence parameters in **(A)** Durc versus Pemc, **(B)** Nivc versus Pemc and **(C)** Nivc versus Atec. Durc, durvalumab plus chemotherapy; Nivc, nivolumab plus chemotherapy; Pemc, pembrolizumab plus chemotherapy; Atec, atezolizumab plus chemotherapy; HR, hazard ratio; INMB, incremental net-monetary benefit; PFS, progression-free survival; PD, progressed disease; OS, overall survival.

We selected three representative pairwise comparisons in the one-way sensitivity analyses. A tornado diagram of the one-way sensitivity analyses is shown in **Figure 2**. The INMBs were substantially sensitive to the cost of the ICIs, and other parameters had medium or small impact on the INMBs.

**Figure 3:**
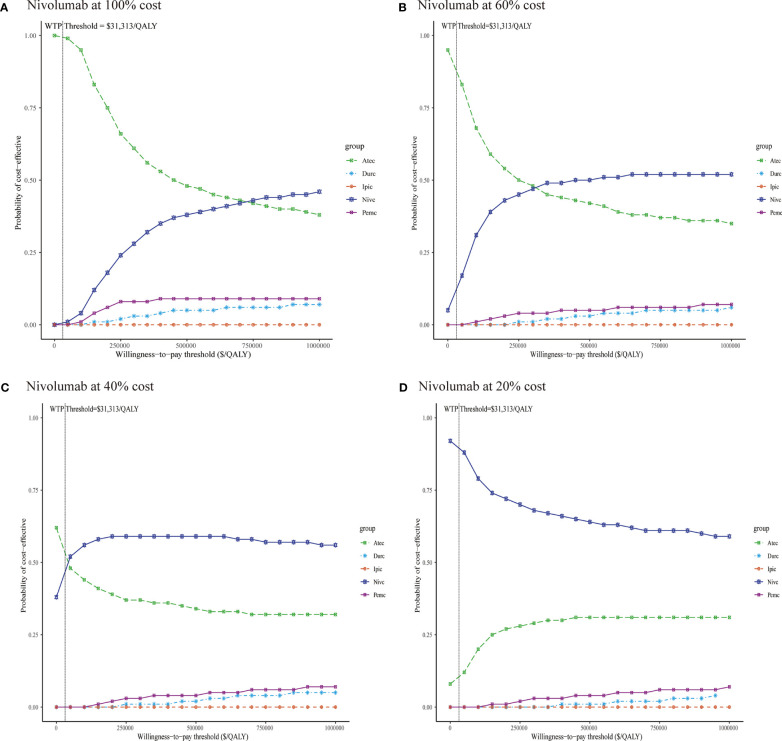
Cost-effectiveness acceptability curves of five immunotherapy plus chemotherapy regimens **(A)** Nivolumab at 100% cost, **(B)** Nivolumab at 60% cost, **(C)** Nivolumab at 40% cost, **(D)** Nivolumab at 20% cost. Durc, durvalumab plus chemotherapy; Atec, atezolizumab plus chemotherapy; Pemc, pembrolizumab plus chemotherapy; Nivc, nivolumab plus chemotherapy; Ipic, ipilimumab plus chemotherapy; QALY, quality-adjusted life year; WTP, willingness-to-pay.

In the probabilistic sensitivity analyses, the cost-effectiveness acceptability curves (**Figure 3A**) demonstrated that the probability of atezolizumab plus chemotherapy being cost-effective was 99.7% at a WTP threshold of $31,313 per QALY gained in China. When the WTP threshold was increased to $740,000/QALY, nivolumab plus chemotherapy was likely to be the most cost-effective option among the five competing regimens.

Concomitant with the decline in the price of nivolumab, the results of the probabilistic sensitivity analyses have changed (**Figures 3B** **–D**). When the price of nivolumab reduced 40 and 60%, atezolizumab plus chemotherapy is still the most preferred option at a WTP threshold of $31,313 per QALY gained in China; nivolumab plus chemotherapy could be considered the most cost-effective strategy when the WTP threshold increased to $320,000/QALY, and $43,000/QALY, respectively. When the price of nivolumab reduced 80%, the combination regimen of nivolumab plus chemotherapy was likely to be the cost-effective first-line treatment for ES-SCLC in China at the WTP threshold of $0/QALY to $100,000/QALY.

## Discussion

Reports of the clinical benefits from ICIs plus chemotherapy as the first-line treatment for ES-SCLC in the clinical trials motivated greatest interest for both oncologists and healthcare decision makers. However, the healthcare cost is increased dramatically due to the high price of the ICIs, so the economic evaluation of the ICIs plus chemotherapy is necessary especially for resource-limited countries such as China. Although previously studies demonstrated that ICIs plus chemotherapy was unlikely to be cost-effective compared with chemotherapy for previously untreated ES-SCLC, the cost-effectiveness between the first-line ICIs plus chemotherapy regimens was not clear yet. So, we conducted the NMA and cost-effectiveness analysis to clear the first treatment option and evaluate the economic outcomes based on the Chinese context. To the best of our knowledge, this is the first study to examine the cost-effectiveness of the five competing, first-line ICIs plus chemotherapy treatment options (atezolizumab plus chemotherapy, durvalumab plus chemotherapy, pembrolizumab plus chemotherapy, nivolumab plus chemotherapy and ipilimumab plus chemotherapy) in China. The findings of the NMA suggested that nivolumab plus chemotherapy was ranked higher than other regimens for previously untreated ES-SCLC. The main findings of the current cost-effectiveness analysis revealed that atezolizumab plus chemotherapy for treating newly diagnosed ES-SCLC could provide relatively more health benefits and less resource consumption, and these findings suggested that atezolizumab plus chemotherapy would be the cost-effective therapeutic approach. The PSA found that the model results were robust. We also conducted the scenarios analyses to explore the potential economic impact of the possible price reduce of nivolumab in the future. The scenarios analyses revealed that when the price of nivolumab reduced 80%, the combination strategy of nivolumab plus chemotherapy would likely to be the most cost-effective first-line option for ES-SCLC of the five competing regimens in China. As the results of previous systematic review have shown (43), because the cost inputs are region-specific, the generalizability and transferability of the results from the cost-effectiveness analysis were limited, so the results and conclusions of the current economic analysis were only applicable to China and not to western countries.

Due to the absence of the direct comparison of the five regimens, we conducted the NMA and use the durvalumab plus chemotherapy regimen as the baseline treatment. One recent analysis evaluated the cost-effectiveness of durvalumab plus chemotherapy versus chemotherapy as the first-line treatment for patients with ES-SCLC based on the CASPIAN trial in Chinese context (21), which reported that the cost of durvalumab plus chemotherapy regimen was similar to the current analysis, and the QALYs were slightly different to the current analysis, that might be caused by the different follow-up times of the CASPIAN trial used in the two analyses. Another study assessed the economic outcomes of atezolizumab plus chemotherapy versus chemotherapy for previously untreated ES-SCLC from the Chinese perspective (22), and the reported QALYs of atezolizumab plus chemotherapy were generally coherent with our study; though the reported costs were high than our analysis, it because that we considered the atezolizumab PAP in the current analysis, and this is the main reason that caused the cost of atezolizumab plus chemotherapy lower than the other four competing strategies. The Chinese government and manufactures adopted a series of measures to improve affordability of the ICIs. Among them, the provision of the PAP is a very important approach. However, we only considered the atezolizumab PAP in our study, because the PAP of durvalumab, pembrolizumab, nivolumab, and ipilimumab were not performed for patients with ES-SCLC, and we will update our analysis when the PAP of them were applicable.

The current economic evaluation has several potential limitations. First, since there are no clinical trials that directly compared the five ICIs plus chemotherapy regimens; the NMA was conducted in our study of an indirect comparison, where the patient characteristics were assumed to be similar. Second, a log-logistic survival model and a gamma survival model were used to estimate the long-time health benefits for durvalumab plus chemotherapy, and the HRs of PFS and OS were used to simulate the long-time health outcomes for other combination regimens of ICIs plus chemotherapy. This approach was another inevitable limitation of the analysis. Third, due to the inconsistency of the subgroup information across the clinical trials, we did not conduct the subgroup analysis of the cost-effectiveness between the five ICIs plus chemotherapy regimens. Fourth, some key model inputs, such as the cost of best supportive care per cycle, were obtained from the published literatures rather than the real-world medical data, sensitivity analyses were performed to explore and minimize the potential uncertainty of the model results. Fifth, certain parameters in the cost-effectiveness analysis were assumed due to the absence of data, and the costs for the management of grade 1/2 treatment-related serious adverse events were excluded from the analysis, although one-way sensitivity analyses performed that only minimal influence of the model results. Finally, the utility values were obtained from the foreign study and that might reflect the health preference of the Chinese population well, although small impact were found in the one-way sensitivity analyses. Despite these limitations, we believe that the current study accurately reflected the clinical conditions of ES-SCLC in China.

## Conclusion

In conclusion, nivolumab plus chemotherapy was the best ranked treatment based on the HRs of PFS and OS compared with other first-line regimens for ES-SCLC. Atezolizumab plus chemotherapy as first-line treatment for patients with ES-SCLC is indicated as a cost-effective option compared with other combination therapy of ICIs plus chemotherapy from the Chinese healthcare system, based on its relatively higher health benefits and lower costs due to the atezolizumab PAP. For the future possible scenarios, when the price of nivolumab reduced 80%, nivolumab plus chemotherapy could be considered the most cost-effective treatment among the five first-line therapies in China.

## Data Availability Statement

The original contributions presented in the study are included in the article/**Supplementary Material**. Further inquiries can be directed to the corresponding authors.

## Author Contributions

FS, WG, SK, and XW were involved in the design of the study. FS, WG, SK, XW, YZ, and BZ collected the data and performed the economic analysis. FS, WG, SK, and XW drafted and critically revised the manuscript. All authors contributed to the article and approved the submitted version.

## Conflict of Interest

The authors declare that the research was conducted in the absence of any commercial or financial relationships that could be construed as a potential conflict of interest.

## Publisher’s Note

All claims expressed in this article are solely those of the authors and do not necessarily represent those of their affiliated organizations, or those of the publisher, the editors and the reviewers. Any product that may be evaluated in this article, or claim that may be made by its manufacturer, is not guaranteed or endorsed by the publisher.

## References

[1] GBD 2017 DALYs and HALE Collaborators. Global, Regional, and National Disability-Adjusted Life-Years (DALYs) for 359 Diseases and Injuries and Healthy Life Expectancy (HALE) for 195 Countries and Territories, 1990-2017: A Systematic Analysis for the Global Burden of Disease Study 2017. Lancet (2018) 392:1859–922. doi: 10.1016/S0140-6736(18)32335-3 PMC6252083 30415748

[2] Siegel RL Miller KD Fuchs HE Jemal A. Cancer Statistics, 2021. CA Cancer J Clin (2021) 71:7–33. doi: 10.3322/caac.21654 33433946

[3] Oronsky B Reid TR Oronsky A Carter CA. What’s New in SCLC? A Review. Neoplasia (2017) 19:842–7. doi: 10.1016/j.neo.2017.07.007 PMC5596356 28888101

[4] Byers LA Rudin CM. Small Cell Lung Cancer: Where Do We Go From Here? Cancer (2015) 121:664–72. doi: 10.1002/cncr.29098 PMC5497465 25336398

[5] Wang S Tang J Sun T Zheng X Li J Sun H. Survival Changes in Patients With Small Cell Lung Cancer and Disparities Between Different Sexes, Socioeconomic Statuses and Ages. Sci Rep (2017) 7:1339. doi: 10.1038/s41598-017-01571-0 28465554 PMC5431017

[6] Pietanza MC Byers LA Minna JD Rudin CM. Small Cell Lung Cancer: Will Recent Progress Lead to Improved Outcomes? Clin Cancer Res (2015) 21:2244–55. doi: 10.1158/1078-0432.CCR-14-2958 PMC4497796 25979931

[7] Rudin CM Ismaila N Hann CL Malhotra N Movsas B Norris K. Treatment of Small-Cell Lung Cancer: American Society of Clinical Oncology Endorsement of the American College of Chest Physicians Guideline. J Clin Oncol (2015) 33:4106–11. doi: 10.1200/JCO.2015.63.7918 26351333

[8] Farago AF Keane FK. Current Standards for Clinical Management of Small Cell Lung Cancer. Transl Lung Cancer Res (2018) 7:69–79. doi: 10.21037/tlcr.2018.01.16 29535913 PMC5835595

[9] Ribas A Wolchok JD. Cancer Immunotherapy Using Checkpoint Blockade. Science (2018) 359:1350–5. doi: 10.1126/science.aar4060 PMC7391259 29567705

[10] Wu B Zhang Q Sun J. Cost-Effectiveness of Nivolumab Plus Ipilimumab as First-Line Therapy in Advanced Renal-Cell Carcinoma. J Immunother Cancer (2018) 6:124. doi: 10.1186/s40425-018-0440-9 30458884 PMC6247499

[11] Lin S Luo S Zhong L Lai S Zeng D Rao X. Cost-Effectiveness of Atezolizumab Plus Chemotherapy for Advanced Non-Small-Cell Lung Cancer. Int J Clin Pharm (2020) 42:1175–83. doi: 10.1007/s11096-020-01076-3 32524512

[12] Peters S Reck M Smit EF Mok T Hellmann MD. How to Make the Best Use of Immunotherapy as First-Line Treatment of Advanced/Metastatic Non-Small-Cell Lung Cancer. Ann Oncol (2019) 30:884–96. doi: 10.1093/annonc/mdz109 30912805

[13] Insinga RP Vanness DJ Feliciano JL Vandormael K Traore S Ejzykowicz F. Cost-Effectiveness of Pembrolizumab in Combination With Chemotherapy Versus Chemotherapy and Pembrolizumab Monotherapy in the First-Line Treatment of Squamous Non-Small-Cell Lung Cancer in the US. Curr Med Res Opin (2019) 35:1241–56. doi: 10.1080/03007995.2019.1571297 30649973

[14] Reck M Luft A Szczesna A Havel L Kim SW Akerley W. Phase III Randomized Trial of Ipilimumab Plus Etoposide and Platinum Versus Placebo Plus Etoposide and Platinum in Extensive-Stage Small-Cell Lung Cancer. J Clin Oncol (2016) 34:3740–8. doi: 10.1200/JCO.2016.67.6601 27458307

[15] Horn L Mansfield AS Szczęsna A Havel L Krzakowski M Hochmair MJ. First-Line Atezolizumab Plus Chemotherapy in Extensive-Stage Small-Cell Lung Cancer. N Engl J Med (2018) 379:2220–9. doi: 10.1056/NEJMoa1809064 30280641

[16] Goldman JW Dvorkin M Chen Y Reinmuth N Hotta K Trukhin D. Durvalumab, With or Without Tremelimumab, Plus Platinum-Etoposide Versus Platinum-Etoposide Alone in First-Line Treatment of Extensive-Stage Small-Cell Lung Cancer (CASPIAN): Updated Results From a Randomised, Controlled, Open-Label, Phase 3 Trial. Lancet Oncol (2021) 22:51–65. doi: 10.1016/S1470-2045(20)30539-8 33285097

[17] Rudin CM Awad MM Navarro A Gottfried M Peters S Csőszi T. Pembrolizumab or Placebo Plus Etoposide and Platinum as First-Line Therapy for Extensive-Stage Small-Cell Lung Cancer: Randomized, Double-Blind, Phase III KEYNOTE-604 Study. J Clin Oncol (2020) 38:2369–79. doi: 10.1200/JCO.20.00793 PMC7474472 32468956

[18] Leal T Wang Y Dowlati A Lewis DA Chen YB Mohindra AR. Randomized Phase II Clinical Trial of Cisplatin/Carboplatin and Etoposide (CE) Alone or in Combination With Nivolumab as Frontline Therapy for Extensive-Stage Small Cell Lung Cancer (ESSCLC): ECOGACRIN Ea5161. J Clin Oncol (2020) 38:9000. doi: 10.1200/JCO.2020.38.15_suppl.9000 PMC12276847 40526078

[19] Pavan A Attili I Pasello G Guarneri V Conte PF Bonanno L. Immunotherapy in Small-Cell Lung Cancer: From Molecular Promises to Clinical Challenges. J Immunother Cancer (2019) 7:205. doi: 10.1186/s40425-019-0690-1 31383005 PMC6683488

[20] Gristina V Galvano A Castellana L Insalaco L Cusenza S Graceffa G. Is There Any Room for PD-1 Inhibitors in Combination With Platinum-Based Chemotherapy as Frontline Treatment of Extensive-Stage Small Cell Lung Cancer? A Systematic Review and Meta-Analysis With Indirect Comparisons Among Subgroups and Landmark Survival Analyses. Ther Adv Med Oncol (2021) 13:17588359211018018. doi: 10.1177/17588359211018018 34646363 PMC8504650

[21] Liu G Kang S. Cost-Effectiveness of Adding Durvalumab to First-Line Chemotherapy for Extensive-Stage Small-Cell Lung Cancer in China. Expert Rev Pharmacoecon Outcomes Res (2021), 1–7. doi: 10.1080/14737167.2021.1888717 33627014

[22] Li LY Wang H Chen X Li WQ Cui JW. First-Line Atezolizumab Plus Chemotherapy in Treatment of Extensive Small Cell Lung Cancer: A Cost-Effectiveness Analysis From China. Chin Med J (Engl) (2019) 132:2790–4. doi: 10.1097/CM9.0000000000000536 PMC6940079 31856049

[23] Cochrane Training. Cochrane RevMan. Available at: https://training.cochrane.org/onlinelearning/core-software-cochrane-reviews/revman (Accessed September 24, 2020).

[24] Chen J Wang J Xu H. Comparison of Atezolizumab, Durvalumab, Pembrolizumab, and Nivolumab as First-Line Treatment in Patients With Extensive-Stage Small Cell Lung Cancer: A Systematic Review and Network Meta-Analysis. Med (Baltimore) (2021) 100:e25180. doi: 10.1097/MD.0000000000025180 PMC8051984 33847617

[25] Su Y Fu J Du J Wu B. First-Line Treatments for Advanced Renal-Cell Carcinoma With Immune Checkpoint Inhibitors: Systematic Review, Network Meta-Analysis and Cost-Effectiveness Analysis. Ther Adv Med Oncol (2020) 12:1758835920950199. doi: 10.1177/1758835920950199 32874210 PMC7436799

[26] Task group of the Chinese guidelines for pharmacoeconomic evaluations. China Guidelines for Pharmacoeconomic Evaluations(2011 Version). China J Pharm Econ (2011) 3:6–9+11-48.

[27] Eichler HG Kong SX Gerth WC Mavros P Jönsson B. Use of Cost-Effectiveness Analysis in Health-Care Resource Allocation Decision-Making: How Are Cost-Effectiveness Thresholds Expected to Emerge? Value Health (2004) 7:518–28. doi: 10.1111/j.1524-4733.2004.75003.x 15367247

[28] Guyot P Ades AE Ouwens MJ Welton NJ. Enhanced Secondary Analysis of Survival Data: Reconstructing the Data From Published Kaplan-Meier Survival Curves. BMC Med Res Methodol (2012) 12:9. doi: 10.1186/1471-2288-12-9 22297116 PMC3313891

[29] Pei R Shi Y Lv S Dai T Zhang F Liu S. Nivolumab vs Pembrolizumab for Treatment of US Patients With Platinum-Refractory Recurrent or Metastatic Head and Neck Squamous Cell Carcinoma: A Network Meta-Analysis and Cost-Effectiveness Analysis. JAMA Netw Open (2021) 4:e218065. doi: 10.1001/jamanetworkopen.2021.8065 33956130 PMC8103222

[30] Gu X Zhang Q Chu YB Zhao YY Zhang YJ Kuo D. Cost-Effectiveness of Afatinib, Gefitinib, Erlotinib and Pemetrexed-Based Chemotherapy as First-Line Treatments for Advanced Non-Small Cell Lung Cancer in China. Lung Cancer (2019) 127:84–9. doi: 10.1016/j.lungcan.2018.11.029 30642557

[31] Zhou K Wen F Zhang P Zhou J Zheng H Sun L. Cost-Effectiveness Analysis of Sensitive Relapsed Small-Cell Lung Cancer Based on JCOG0605 Trial. Clin Transl Oncol (2018) 20:768–74. doi: 10.1007/s12094-017-1787-y 29098555

[32] Nafees B Stafford M Gavriel S Bhalla S Watkins J. Health State Utilities for Non Small Cell Lung Cancer. Health Qual Life Outcomes (2008) 6:84. doi: 10.1186/1477-7525-6-84 18939982 PMC2579282

[33] Wu B Gu X Zhang Q Xie F. Cost-Effectiveness of Osimertinib in Treating Newly Diagnosed, Advanced EGFR-Mutation-Positive Non-Small Cell Lung Cancer. Oncologist (2019) 24:349–57. doi: 10.1634/theoncologist.2018-0150 PMC6519771 30257889

[34] Lu S Ye M Ding L Tan F Fu J Wu B. Cost-Effectiveness of Gefitinib, Icotinib, and Pemetrexed-Based Chemotherapy as First-Line Treatments for Advanced Non-Small Cell Lung Cancer in China. Oncotarget (2017) 8:9996–10006. doi: 10.18632/oncotarget.14310 28036283 PMC5354787

[35] Zhou K Jiang C Li Q. Cost-Effectiveness Analysis of Pembrolizumab Monotherapy and Chemotherapy in the Non-Small-Cell Lung Cancer With Different PD-L1 Tumor Proportion Scores. Lung Cancer (2019) 136:98–101. doi: 10.1016/j.lungcan.2019.08.028 31476529

[36] Zhang PF Xie D Li Q. Cost-Effectiveness Analysis of Nivolumab in the Second-Line Treatment for Advanced Esophageal Squamous Cell Carcinoma. Future Oncol (2020) 17:1189–98. doi: 10.2217/fon-2019-0821 32407173

[37] Yang Z Zhu Y Xiang G Hua T Ni J Zhao J. First-Line Atezolizumab Plus Chemotherapy in Advanced Non-Squamous Non-Small Cell Lung Cancer: A Cost-Effectiveness Analysis From China. Expert Rev Pharmacoecon Outcomes Res (2021) 21:1061–7. doi: 10.1080/14737167.2021.1899813 33682554

[38] Lu S Zhang J Ye M Wang B Wu B. Economic Analysis of ALK Testing and Crizotinib Therapy for Advanced Non-Small-Cell Lung Cancer. Pharmacogenomics (2016) 17:985–94. doi: 10.2217/pgs-2016-0017 27266545

[39] Liu XY Chen W. Pharmacoeconomic Evaluation of Osimertinib in the First-Line Treatment of Locally Advanced or Metastatic NSCLC With an EGFR Mutation. World Clin Drugs (2021) 42:135–42. doi: 10.13683/j.wph.2021.02.010

[40] Hao X Shen A Wu B. Cost-Effectiveness of Nivolumab Plus Ipilimumab as First-Line Therapy in Advanced Non-Small-Cell Lung Cancer. Front Pharmacol (2021) 12:573852. doi: 10.3389/fphar.2021.573852 34290602 PMC8287729

[41] Nafees B Lloyd AJ Dewilde S Rajan N Lorenzo M. Health State Utilities in Non-Small Cell Lung Cancer: An International Study. Asia Pac J Clin Oncol (2017) 13:e195–203. doi: 10.1111/ajco.12477 26990789

[42] Briggs AH Weinstein MC Fenwick EA Karnon J Sculpher MJ Paltiel AD. Model Parameter Estimation and Uncertainty Analysis: A Report of the ISPOR-SMDM Modeling Good Research Practices Task Force Working Group-6. Med Decis Making (2012) 32:722–32. doi: 10.1177/0272989X12458348 22990087

[43] Petrou P. A Systematic Review of Economic Evaluations of Tyrosine Kinase Inhibitors of Vascular Endothelial Growth Factor Receptors, Mammalian Target of Rapamycin Inhibitors and Programmed Death-1 Inhibitors in Metastatic Renal Cell Cancer. Expert Rev Pharmacoecon Outcomes Res (2018) 18:255–65. doi: 10.1080/14737167.2018.1439740 29448845

