# Supplementary material for: First-Line Treatments for Extensive-Stage Small-Cell Lung Cancer With Immune Checkpoint Inhibitors Plus Chemotherapy: A Network Meta-Analysis and Cost-Effectiveness Analysis
Source: Front Oncol. 2022 Jan 19;11:740091. doi: 10.3389/fonc.2021.740091 (PMC8807476; doi:10.3389/fonc.2021.740091)
Supplement: Supplementary file 1 [file DataSheet_1.docx]

Supplementary Figures

**Supplementary Figure 1 |** The replicated Kaplan-Meier PFS and OS curves of atezolizumab plus chemotherapy regimen in IMpower133 trial. PFS, progression-free survival; OS, overall survival; Atec, atezolizumab plus chemotherapy.

**Supplementary Figure 2 |** The replicated Kaplan-Meier PFS and OS curves of durvalumab plus chemotherapy regimen in CASPIAN trial. PFS, progression-free survival; OS, overall survival; Durc, durvalumab plus chemotherapy.

**Supplementary Figure 3 |** The replicated Kaplan-Meier PFS and OS curves of pembrolizumab plus chemotherapy regimen in KEYNOTE-604 trial. PFS, progression-free survival; OS, overall survival; Pemc, pembrolizumab plus chemotherapy.

**Supplementary Figure 4 |** The replicated Kaplan-Meier PFS and OS curves of ipilimumab plus chemotherapy regimen in CA184-156 trial. PFS, progression-free survival; OS, overall survival; Ipic, ipilimumab plus chemotherapy.

**Supplementary Figure 5 |** Flowchart of study selection.

**Supplementary Figure 6 |** Model schematic for the network meta-analysis. Atec, atezolizumab plus chemotherapy; Durc, durvalumab plus chemotherapy; Pemc, pembrolizumab plus chemotherapy; Nivc, nivolumab plus chemotherapy; Ipic, ipilimumab plus chemotherapy.

**Supplementary Figure 7 |** Risk of bias summary.

**Supplementary Figure 8 |** Results of network meta-analysis. PFS, progression-free survival; OS, overall survival; Atec, atezolizumab plus chemotherapy; Durc, durvalumab plus chemotherapy; Pemc, pembrolizumab plus chemotherapy; Nivc, nivolumab plus chemotherapy; Ipic, ipilimumab plus chemotherapy; Chem, chemotherapy.


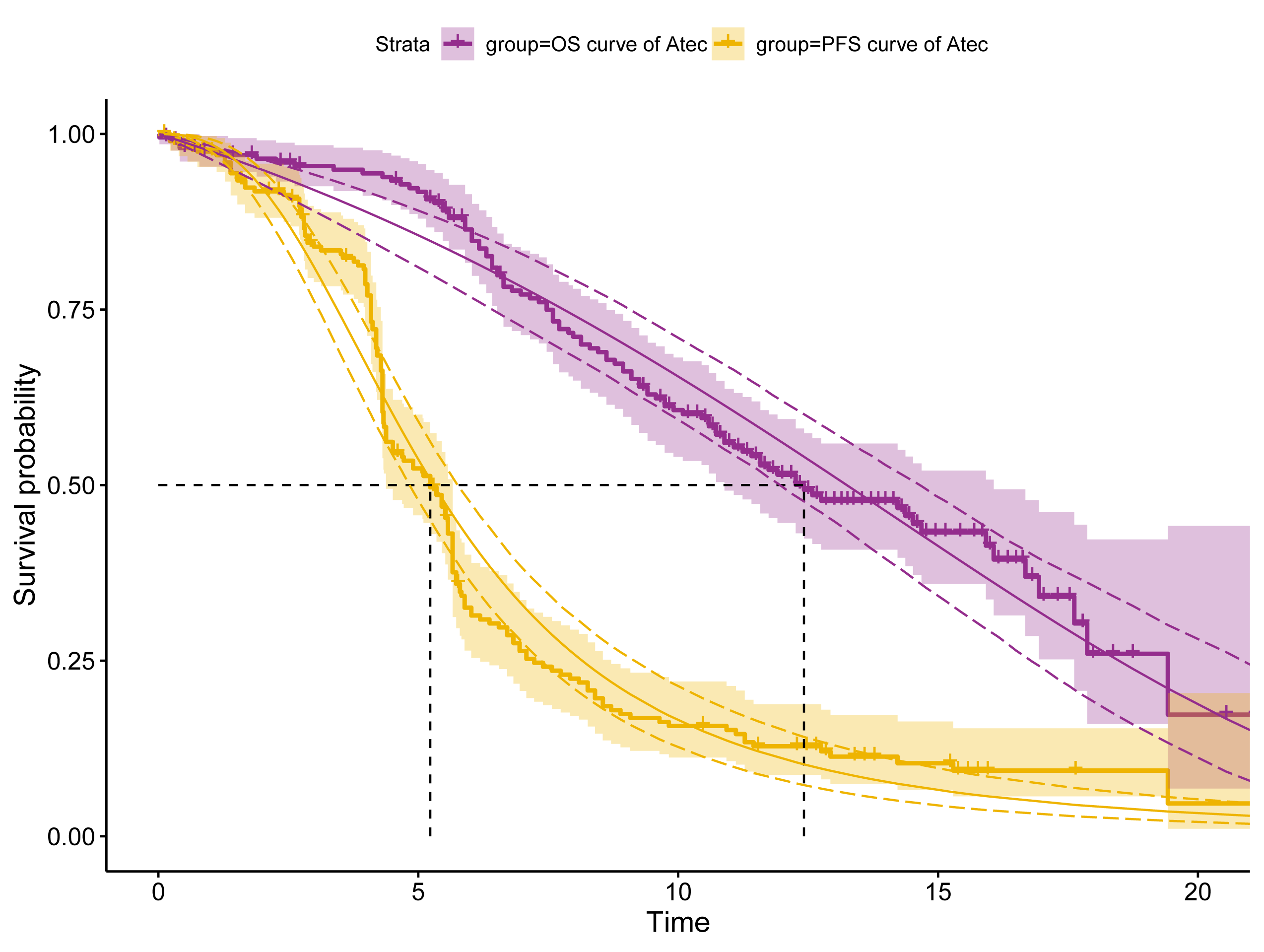


**Supplementary Figure 1** | The replicated Kaplan-Meier PFS and OS curves of atezolizumab plus chemotherapy regimen in IMpower133 trial. PFS, progression-free survival; OS, overall survival; Atec, atezolizumab plus chemotherapy.


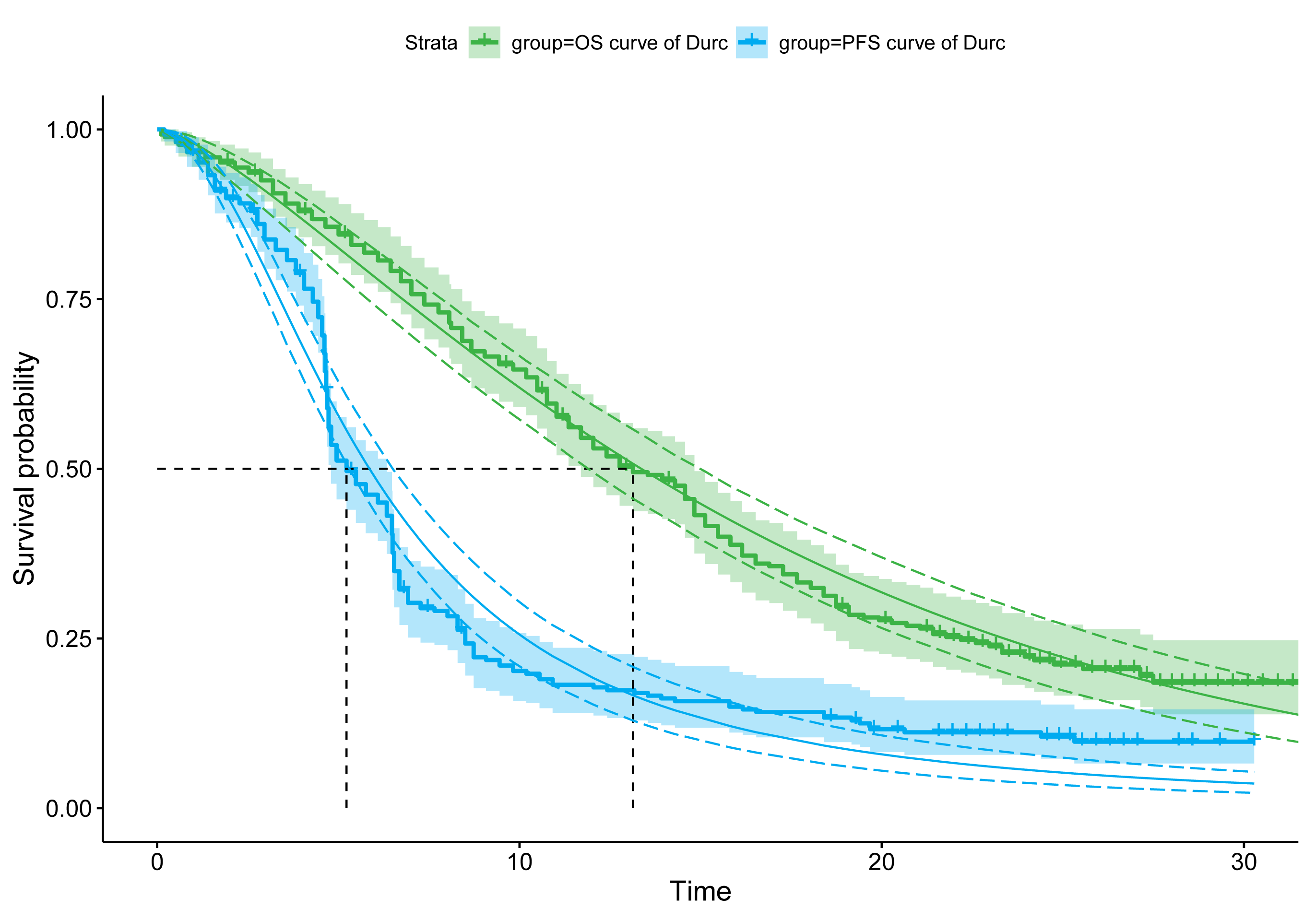


**Supplementary Figure 2 |** The replicated Kaplan-Meier PFS and OS curves of durvalumab plus chemotherapy regimen in CASPIAN trial. PFS, progression-free survival; OS, overall survival; Durc, durvalumab plus chemotherapy.

**Supplementary Figure 3 |** The replicated Kaplan-Meier PFS and OS curves of pembrolizumab plus chemotherapy regimen in KEYNOTE-604 trial. PFS, progression-free survival; OS, overall survival; Pemc, pembrolizumab plus chemotherapy.**
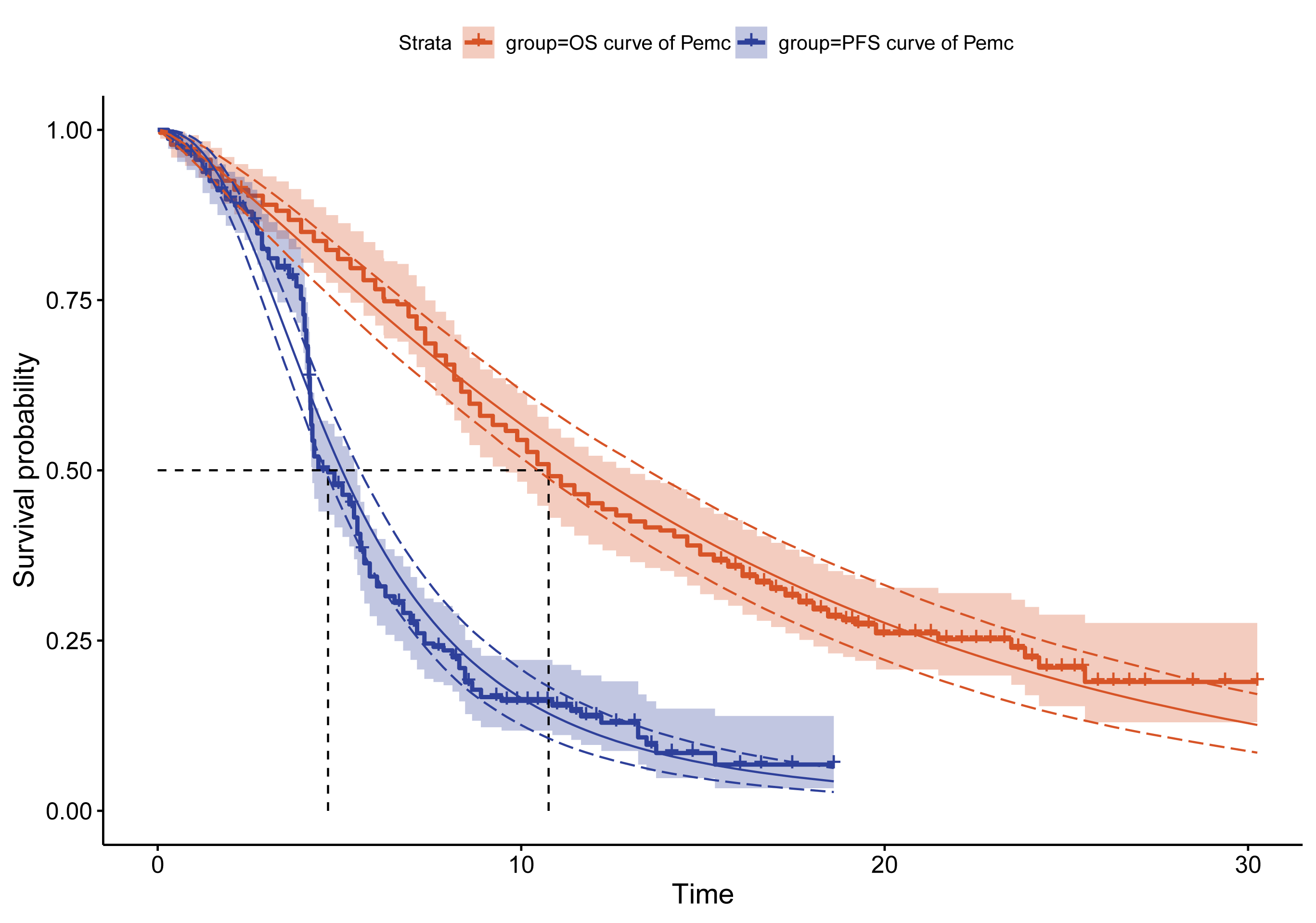
**

**Supplementary Figure 4 |** The replicated Kaplan-Meier PFS and OS curves of ipilimumab plus chemotherapy regimen in CA184-156 trial. PFS, progression-free survival; OS, overall survival; Ipic, ipilimumab plus chemotherapy.
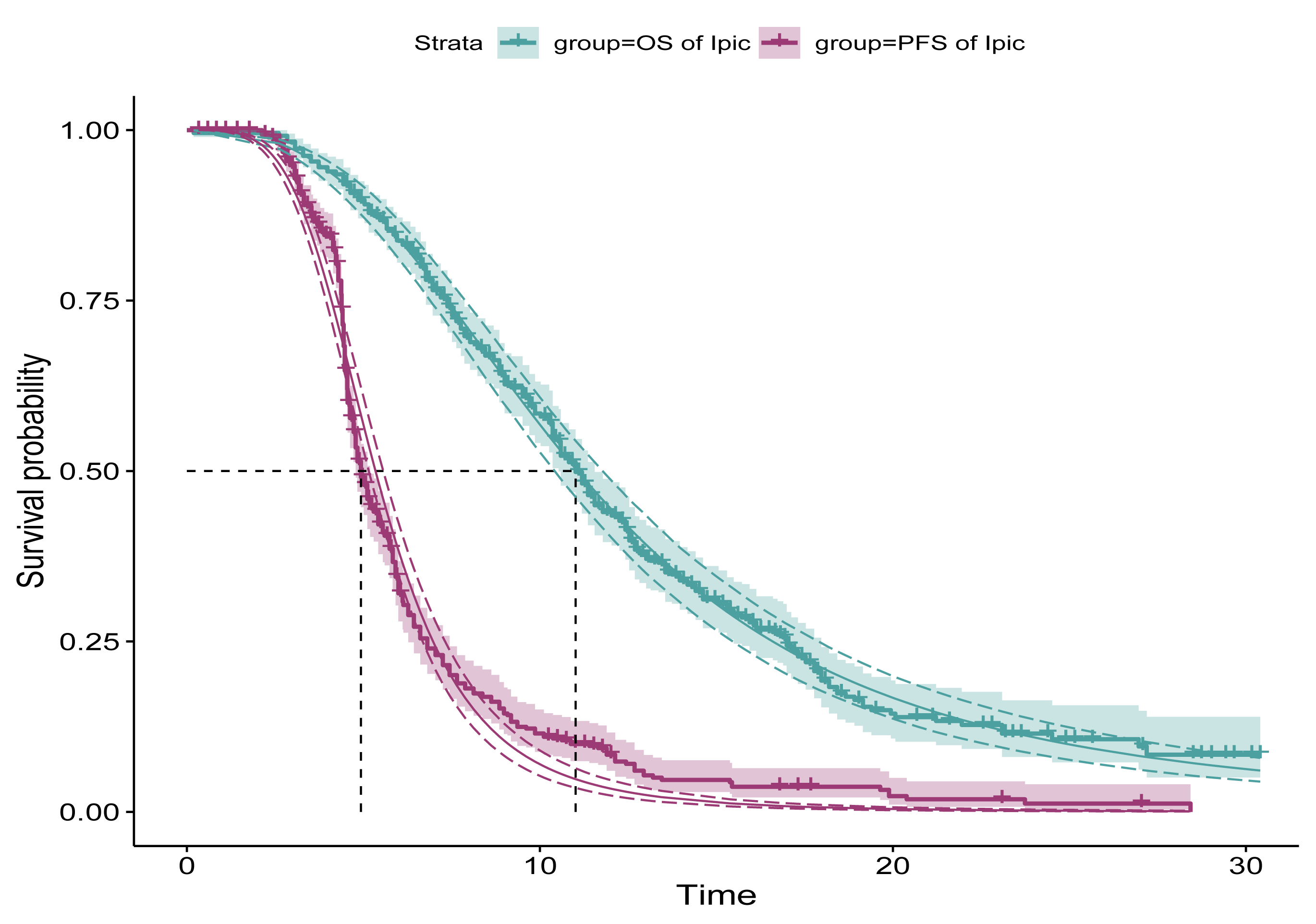


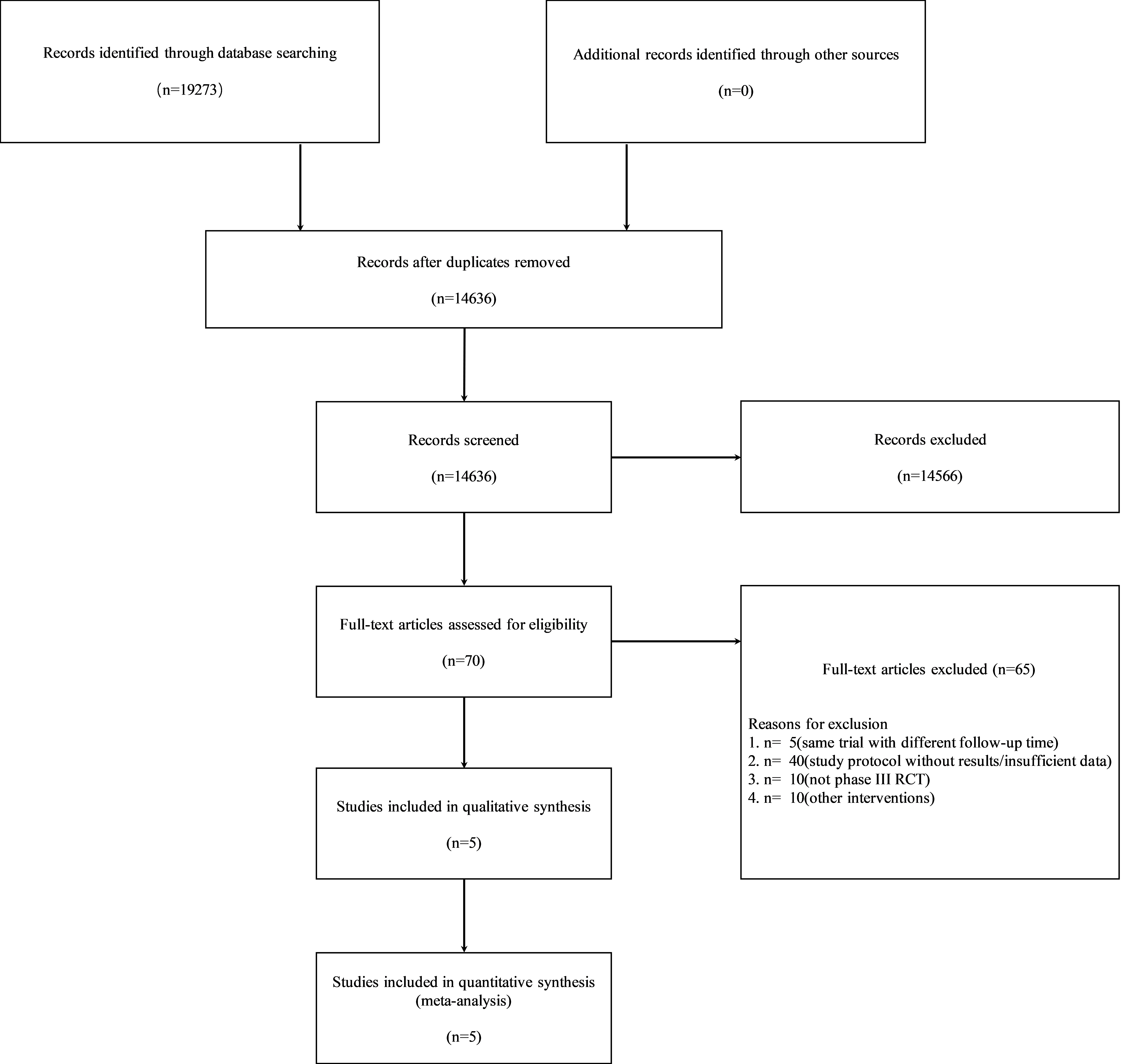


**Supplementary Figure 5 |** Flowchart of study selection.


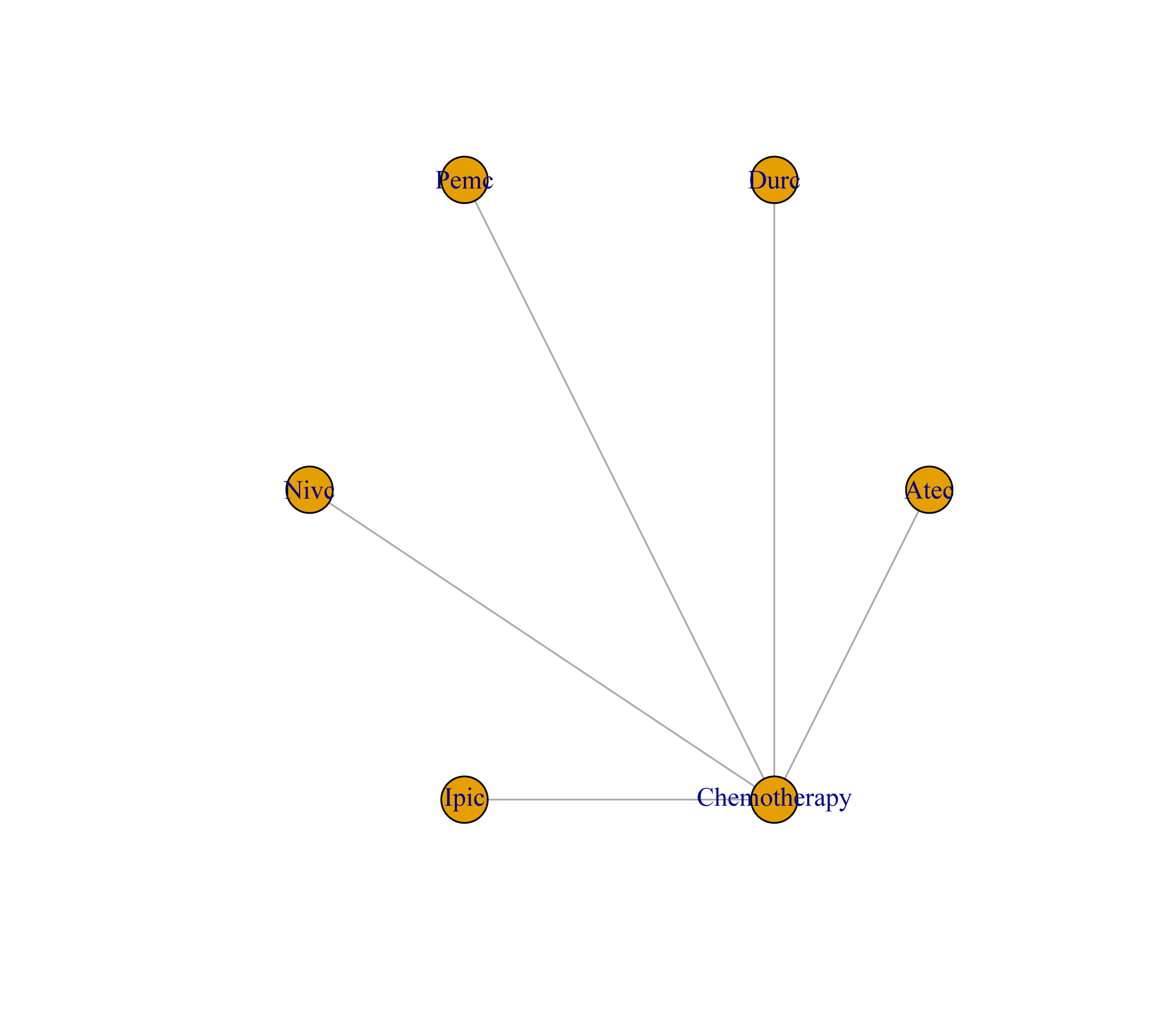


**Supplementary Figure 6 |** Model schematic for the network meta-analysis. Atec, atezolizumab plus chemotherapy; Durc, durvalumab plus chemotherapy; Pemc, pembrolizumab plus chemotherapy; Nivc, nivolumab plus chemotherapy; Ipic, ipilimumab plus chemotherapy.


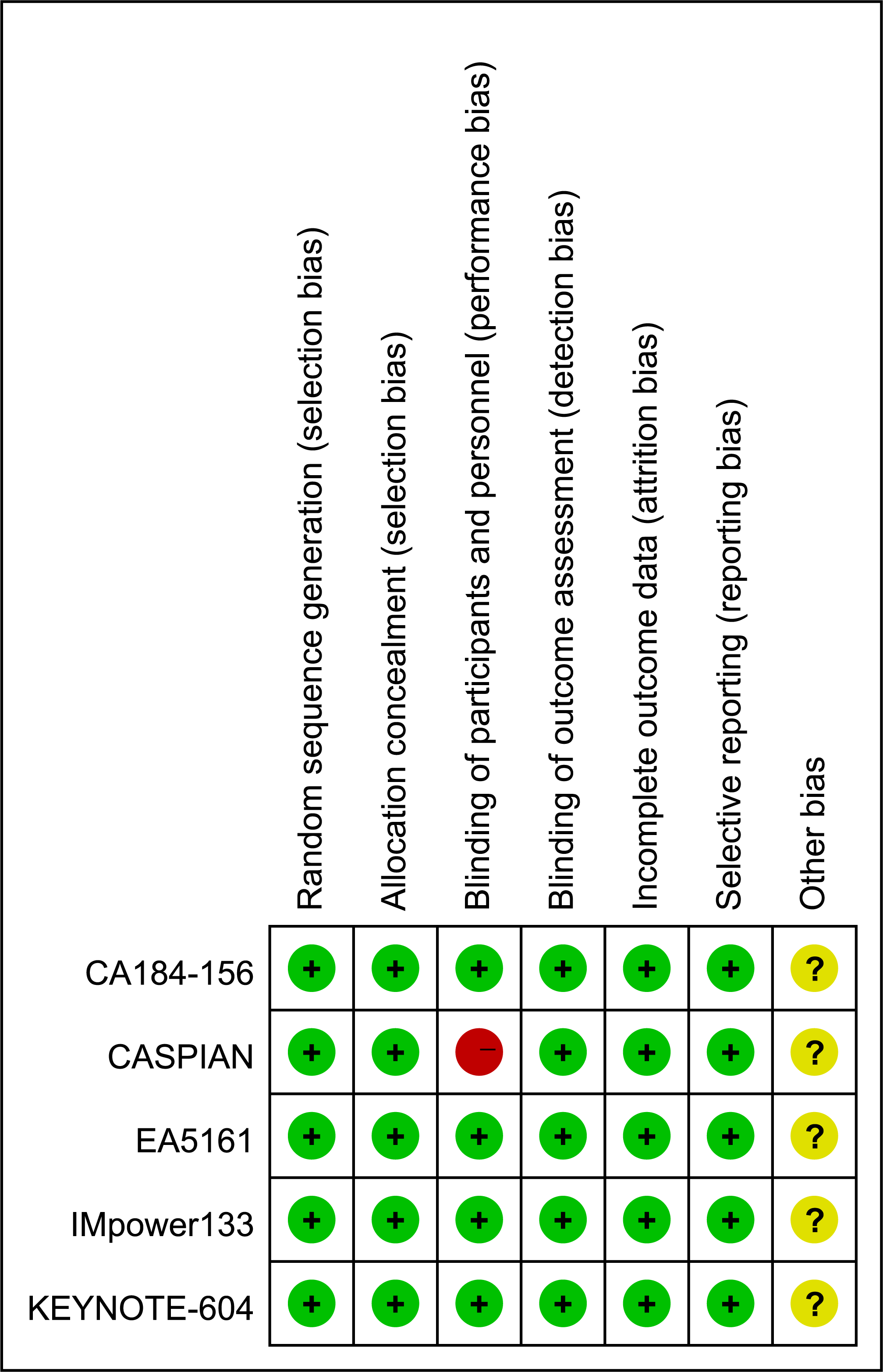


**Supplementary Figure 7 |** Risk of bias summary.


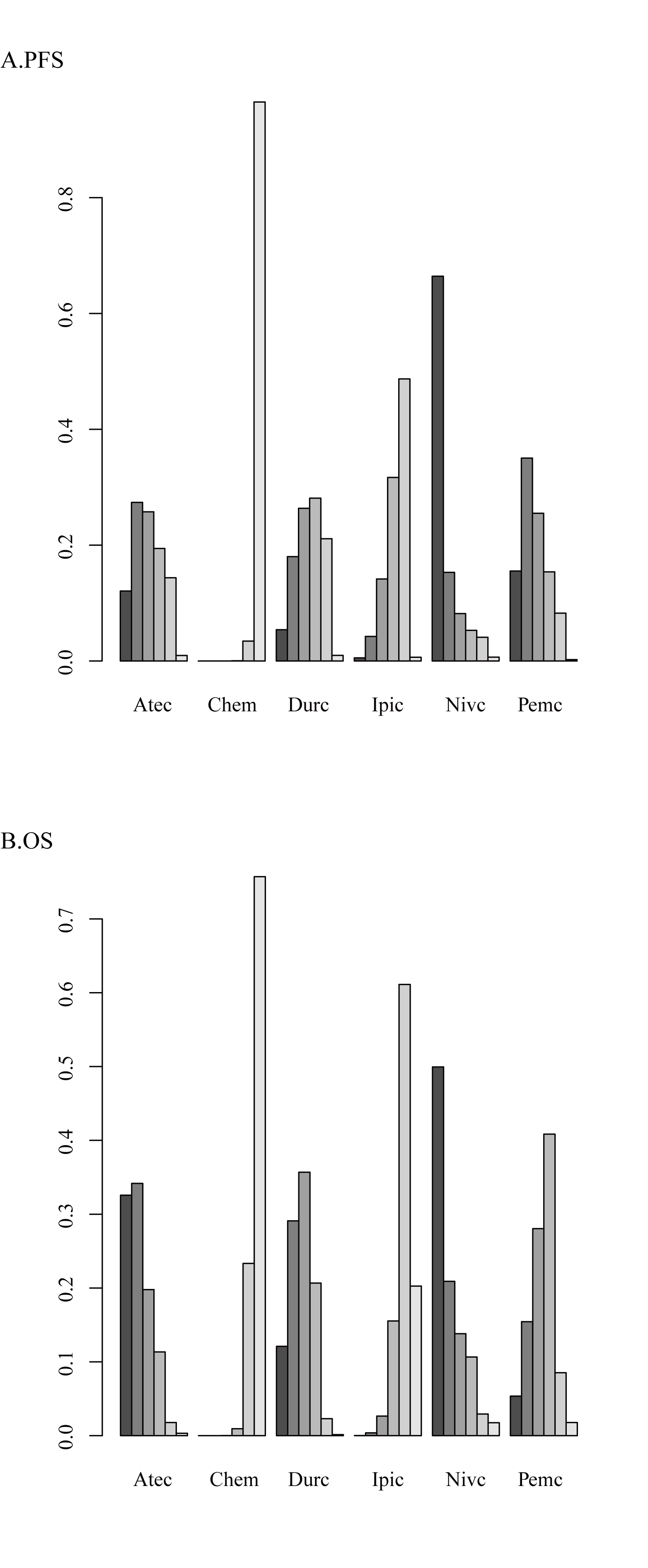


**Supplementary Figure 8 |** Results of network meta-analysis. PFS, progression-free survival; OS, overall survival; Atec, atezolizumab plus chemotherapy; Durc, durvalumab plus chemotherapy; Pemc, pembrolizumab plus chemotherapy; Nivc, nivolumab plus chemotherapy; Ipic, ipilimumab plus chemotherapy; Chem, chemotherapy.
