# Supplementary material for: First-Line Treatments for Extensive-Stage Small-Cell Lung Cancer With Immune Checkpoint Inhibitors Plus Chemotherapy: A Network Meta-Analysis and Cost-Effectiveness Analysis
Source: Front Oncol. 2022 Jan 19;11:740091. doi: 10.3389/fonc.2021.740091 (PMC8807476; doi:10.3389/fonc.2021.740091)
Supplement: Supplementary file 2 [file DataSheet_2.docx]

Supplementary Tables and Files

**Supplementary Table 1 |** Summary of statistical goodness-of-fit of Kaplan-Meier curves in IMpower133, CASPIAN, KEYNOTE-604, and CA184-156 trials.

**Supplementary Table 2 |** Inclusion and exclusion criteria of the network meta-analysis.

**Supplementary Table 3 |** Characteristics of the randomized controlled trials included in the network meta-analysis.

**Supplementary Table 4 |** PRISMA NMA checklist of items to include when reporting a systematic review involving a network meta-analysis.

**Supplementary File 1 |** Search strategies for PubMed, EMBASE and Cochrane database of network meta-analysis.

**Supplementary Table 1** **|** Summary of statistical goodness-of-fit of Kaplan-Meier curves in IMpower133, CASPIAN, KEYNOTE-604, and CA184-156 trials.

|  | Exponential | Gamma | Weibull | Log-normal | Log-logistic | Gompertz |
| --- | --- | --- | --- | --- | --- | --- |
| PFS curve of atezolizumab plus chemotherapy | | | | | | |
| AIC | 986.61 | 936.05 | 946.21 | 943.17 | 916.81 | 975.59 |
| OS curve of atezolizumab plus chemotherapy | | | | | | |
| AIC | 827.77 | 809.42 | 805.47 | 843.02 | 810.03 | 804.74 |
| PFS curve of durvalumab plus chemotherapy | | | | | | |
| AIC | 1481.524 | 1468.318 | 1478.315 | 1432.634 | 1409.15* | 1477.489 |
| OS curve of durvalumab plus chemotherapy | | | | | | |
| AIC | 1592.983 | 1578.29* | 1578.307 | 1608.434 | 1580.253 | 1586.447 |
| PFS curve of pembrolizumab plus chemotherapy | | | | | | |
| AIC | 1089.657 | 1038.537 | 1046.458 | 1048.104 | 1025.688 | 1075.534 |
| OS curve of pembrolizumab plus chemotherapy | | | | | | |
| AIC | 1285.142 | 1279.032 | 1279.622 | 1295.46 | 1279.162 | 1284.386 |
| PFS curve of ipilimumab plus chemotherapy | | | | | | |
| AIC | 2420.877 | 2065.004 | 2177.638 | 1981.21 | 1953.482 | 2348.948 |
| OS curve of ipilimumab plus chemotherapy | | | | | | |
| AIC | 2593.761 | 2417.307 | 2432.033 | 2436.302 | 2401.398 | 2504.284 |

**, best fitted model; AIC, Akaike information criterion; PFS, progression-free survival; OS, overall survival.*

**Supplementary Table 2 |** Inclusion and exclusion criteria of the network meta-analysis

| Literature characteristics | Inclusion criteria | Exclusion criteria |
| --- | --- | --- |
| Population | Adult patients with previously untreated extensive-stage small-cell lung cancer | Patients < 18years old |
|  |  | Healthy subjects |
| Intervention | Atezolizumab plus chemotherapy | Interventions not for first-line treatment |
|  | Pembrolizumab plus chemotherapy |  |
|  | Durvalumab plus chemotherapy |  |
|  | Nivolumab plus chemotherapy |  |
|  | Ipilimumab plus chemotherapy |  |
| Comparators | Chemotherapy | Non-pharmaceutical treatments |
| Outcomes | Progression-free survival | Patient-reported outcomes |
|  | Overall survival | Biomarker outcomes |
| Study design | Randomized controlled trial (RCT) | Non-RCT |
| Language | English | Non-English |

**Supplementary Table 3 |** Characteristics of the randomized controlled trials included in the network meta-analysis

| Study | Author | Year | Study design | Sample size | Patient | Intervention | Control | HR of PFS (95% CI) | HR of OS (95% CI) |
| --- | --- | --- | --- | --- | --- | --- | --- | --- | --- |
| CA184-156 | Reck M. et al | 2016 | RCT | 954 | ES-SCLC | Ipic | Chemotherapy | 0.85 (0.75-0.97) | 0.94 (0.81-1.09) |
| IMpower133 | Horn L. et al | 2018 | RCT | 403 | ES-SCLC | Atec | Chemotherapy | 0.77 (0.62-0.96) | 0.7 (0.54-0.91) |
| EA5161 | Leal T, et al | 2020 | RCT | 180 | ES-SCLC | Nivc | Chemotherapy | 0.65 (0.46-0.91) | 0.67 (0.46-0.98) |
| KEYNOTE-604 | Rudin CM. et al | 2020 | RCT | 453 | ES-SCLC | Pemc | Chemotherapy | 0.75 (0.61-0.91) | 0.8 (0.64-0.98) |
| CASPIAN | Goldman JW. et al | 2021 | RCT | 537 | ES-SCLC | Durc | Chemotherapy | 0.8 (0.66-0.96) | 0.75 (0.62-0.91) |

*HR, hazard ratio; PFS, progression-free survival; OS, overall survival; Ipic, ipilimumab plus chemotherapy; Atec, atezolizumab plus chemotherapy; Nivc, nivolumab plus chemotherapy; Pemc, pembrolizumab plus chemotherapy; Nivc, nivolumab plus chemotherapy; Durc, durvalumab plus chemotherapy; RCT, randomized controlled trial.*

**Supplementary Table 4 |** PRISMA NMA checklist of items to include when reporting a systematic review involving a network meta-analysis.

| **Section/Topic** | **Item** | **Checklist Item** | **Reported** |
| --- | --- | --- | --- |
|  | **#** |  | **on Page #** |
| **TITLE** |  |  |  |
|  |  |  |  |
| Title | 1 | Identify the report as a systematic review *incorporating a* | 1 |
|  |  | *network meta-analysis (or related form of meta-analysis).* |  |
| **ABSTRACT** |  |  |  |
| Structured | 2 | Provide a structured summary including, as applicable: | 2 |
| summary |  | **Background:** main objectives |  |
|  |  | **Methods:** data sources; study eligibility criteria, participants, |  |
|  |  | and interventions; study appraisal; and *synthesis methods,* |  |
|  |  | *such as network meta-analysis.* |  |
|  |  | **Results:** number of studies and participants identified; |  |
|  |  | summary estimates with corresponding confidence/credible |  |
|  |  | intervals; *treatment rankings may also be discussed. Authors* |  |
|  |  | *may choose to summarize pairwise comparisons against a* |  |
|  |  | *chosen treatment included in their analyses for brevity.* |  |
|  |  | **Discussion/Conclusions:** limitations; conclusions and |  |
|  |  | implications of findings. |  |
|  |  | **Other:** primary source of funding; systematic review |  |
|  |  | registration number with registry name. |  |
| **INTRODUCTION** |  |  |  |
|  |  |  |  |
| Rationale | 3 | Describe the rationale for the review in the context of what is | 4 |
|  |  | already known*, including mention of why a network meta-* |  |
|  |  | *analysis has been conducted.* |  |
| Objectives | 4 | Provide an explicit statement of questions being addressed, | 4 |
|  |  | with reference to participants, interventions, comparisons, |  |
|  |  | outcomes, and study design (PICOS). |  |
| **METHODS** |  |  |  |
|  |  |  |  |
| Protocol and | 5 | Indicate whether a review protocol exists and if and where it | Not |
| registration |  | can be accessed (e.g., Web address); and, if available, provide | applicable |
|  |  | registration information, including registration number. |  |
| Eligibility criteria | 6 | Specify study characteristics (e.g., PICOS, length of follow-up) | Supplementary |
|  |  | and report characteristics (e.g., years considered, language, | table 2 |
|  |  | publication status) used as criteria for eligibility, giving |  |
|  |  | rationale. *Clearly describe eligible treatments included in the* |  |
|  |  | *treatment network, and note whether any have been clustered* |  |
|  |  | *or merged into the same node (with justification).* |  |
| Information sources | 7 | Describe all information sources (e.g., databases with dates of | 4 |
|  |  | coverage, contact with study authors to identify additional |  |
|  |  | studies) in the search and date last searched. |  |
| Search | 8 | Present full electronic search strategy for at least one database, | Supplementary |
|  |  | including any limits used, such that it could be repeated. | file 1 |
| Study selection | 9 | State the process for selecting studies (i.e., screening, | 4-5 |
|  |  | eligibility, included in systematic review, and, if applicable, |  |
|  |  |  |  |

|  |  | included in the meta-analysis). |  |  |
| --- | --- | --- | --- | --- |
|  |  |  |  |  |
| Data collection | 10 | Describe method of data extraction from reports (e.g., piloted |  | 5 |
| process |  | forms, independently, in duplicate) and any processes for |  |  |
|  |  | obtaining and confirming data from investigators. |  |  |
| Data items | 11 | List and define all variables for which data were sought (e.g., |  | 5 |
|  |  | PICOS, funding sources) and any assumptions and |  |  |
|  |  | simplifications made. |  |  |
| **Geometry of the** | **S1** | Describe methods used to explore the geometry of the |  | 5 |
| **network** |  | treatment network under study and potential biases related to it. |  |  |
|  |  | This should include how the evidence base has been |  |  |
|  |  | graphically summarized for presentation, and what |  |  |
|  |  | characteristics were compiled and used to describe the evidence |  |  |
|  |  | base to readers. |  |  |
| Risk of bias within | 12 | Describe methods used for assessing risk of bias of individual |  | 5 |
| individual studies |  | studies (including specification of whether this was done at the |  |  |
|  |  | study or outcome level), and how this information is to be used |  |  |
|  |  | in any data synthesis. |  |  |
| Summary measures | 13 | State the principal summary measures (e.g., risk ratio, |  | 5 |
|  |  | difference in means). *Also describe the use of additional* |  |  |
|  |  | *summary measures assessed, such as treatment rankings and* |  |  |
|  |  | *surface under the cumulative ranking curve (SUCRA) values,* |  |  |
|  |  | *as well as modified approaches used to present summary* |  |  |
|  |  | *findings from meta-analyses.* |  |  |
| Planned methods of | 14 | Describe the methods of handling data and combining results of |  | 5 |
| analysis |  | studies for each network meta-analysis. This should include, |  |  |
|  |  | but not be limited to: |  |  |
|  |  | • *Handling of multi-arm trials;* |  |  |
|  |  | • *Selection of variance structure;* |  |  |
|  |  | • *Selection of prior distributions in Bayesian analyses;* |  |  |
|  |  | *and* |  |  |
|  |  | •*Assessment of model fit.* |  |  |
| **Assessment of** | **S2** | Describe the statistical methods used to evaluate the agreement |  | 5 |
| **Inconsistency** |  | of direct and indirect evidence in the treatment network(s) |  |  |
|  |  | studied. Describe efforts taken to address its presence when |  |  |
|  |  | found. |  |  |
| Risk of bias across | 15 | Specify any assessment of risk of bias that may affect the | 7 | Not |
| studies |  | cumulative evidence (e.g., publication bias, selective reporting |  | applicable |
|  |  | within studies). |  |  |
| Additional analyses | 16 | Describe methods of additional analyses if done, indicating |  | Not |
|  |  | which were pre-specified. This may include, but not be limited |  | applicable |
|  |  | to, the following: |  |  |

• Sensitivity or subgroup analyses;

• Meta-regression analyses;

• *Alternative formulations of the treatment network; and*

• *Use of alternative prior distributions for Bayesian*

*analyses (if applicable).*

| **RESULTS†** |  |  |  |
| --- | --- | --- | --- |
|  |  |  |  |
| Study selection | 17 | Give numbers of studies screened, assessed for eligibility, and | Supplementary |
|  |  | included in the review, with reasons for exclusions at each | figure 5 |
|  |  | stage, ideally with a flow diagram. |  |
| **Presentation of** | **S3** | Provide a network graph of the included studies to enable | Supplementary |
| **network structure** |  | visualization of the geometry of the treatment network. | figure 6 |
| **Summary of** | **S4** | Provide a brief overview of characteristics of the treatment | Not |
| **network geometry** |  | network. This may include commentary on the abundance of | applicable |
|  |  | trials and randomized patients for the different interventions |  |
|  |  | and pairwise comparisons in the network, gaps of evidence in |  |
|  |  | the treatment network, and potential biases reflected by the |  |
|  |  | network structure. |  |
| Study | 18 | For each study, present characteristics for which data were | Supplementary |
| characteristics |  | extracted (e.g., study size, PICOS, follow-up period) and | table 3 |
|  |  | provide the citations. |  |
| Risk of bias within | 19 | Present data on risk of bias of each study and, if available, any | Supplementary |
| studies |  | outcome level assessment. | figure 7 |
|  |  |  |  |
| Results of | 20 | For all outcomes considered (benefits or harms), present, for | 9 |
| individual studies |  | each study: 1) simple summary data for each intervention |  |
|  |  | group, and 2) effect estimates and confidence intervals. |  |
|  |  | *Modified approaches may be needed to deal with information* |  |
|  |  | *from larger networks.* |  |
| Synthesis of results | 21 | Present results of each meta-analysis done, including | Table 2 |
|  |  | confidence/credible intervals. *In larger networks, authors may* | Supplementary |
|  |  | *focus on comparisons versus a particular comparator (e.g.* | figure 8 |
|  |  | *placebo or standard care), with full findings presented in an* |  |
|  |  | *appendix. League tables and forest plots may be considered to* |  |
|  |  | *summarize pairwise comparisons.* If additional summary |  |
|  |  | measures were explored (such as treatment rankings), these |  |
|  |  | should also be presented. |  |
| **Exploration for** | **S5** | Describe results from investigations of inconsistency. This may | Not |
| **inconsistency** |  | include such information as measures of model fit to compare | applicable |
|  |  | consistency and inconsistency models, *P* values from statistical |  |
|  |  | tests, or summary of inconsistency estimates from different |  |
|  |  | parts of the treatment network. |  |
| Risk of bias across | 22 | Present results of any assessment of risk of bias across studies | Not |
| studies |  | for the evidence base being studied. | applicable |
|  |  |  |  |
| Results of | 23 | Give results of additional analyses, if done (e.g., sensitivity or | Not |
| additional analyses |  | subgroup analyses, meta-regression analyses*, alternative* | applicable |
|  |  | *network geometries studied, alternative choice of prior* |  |
|  |  | *distributions for Bayesian analyses,* and so forth). |  |
| **DISCUSSION** |  |  |  |
|  |  |  |  |
| Summary of | 24 | Summarize the main findings, including the strength of | 11 |
| evidence |  | evidence for each main outcome; consider their relevance to |  |
|  |  | key groups (e.g., healthcare providers, users, and policy- |  |
|  |  | makers). |  |
| Limitations | 25 | Discuss limitations at study and outcome level (e.g., risk of | 13 |
|  |  | bias), and at review level (e.g., incomplete retrieval of |  |
|  |  | identified research, reporting bias). *Comment on the validity of* |  |
|  |  | *the assumptions, such as transitivity and consistency. Comment* |  |

|  |  | *on any concerns regarding network geometry (e.g., avoidance* |  |  |
| --- | --- | --- | --- | --- |
|  |  | *of certain comparisons).* |  |  |
|  |  |  |  |  |
| Conclusions | 26 | Provide a general interpretation of the results in the context of |  | 14 |
|  |  | other evidence, and implications for future research. |  |  |
|  |  |  |  |  |
| **FUNDING** |  |  |  |  |
| Funding | 27 | Describe sources of funding for the systematic review and other |  | 14-15 |
|  |  | support (e.g., supply of data); role of funders for the systematic |  |  |
|  |  | review. This should also include information regarding whether |  |  |
|  |  | funding has been received from manufacturers of treatments in |  |  |

the network and/or whether some of the authors are content experts with professional conflicts of interest that could affect use of treatments in the network.

PICOS = population, intervention, comparators, outcomes, study design.

* Text in italics indicates wording specific to reporting of network meta-analyses that has been added to guidance from the PRISMA statement.

† Authors may wish to plan for use of appendices to present all relevant information in full detail for items in this section.

**Supplementary File 1 |** Search strategies for PubMed, EMBASE and Cochrane database of network meta-analysis.

PubMed: 4130 results

(((((((((((((((((((((((((((((((((((((((((((((((("pembrolizumab" [Supplementary Concept]) OR (SCH-900475[Title/Abstract])) OR (Keytruda[Title/Abstract])) OR (MK-3475[Title/Abstract])) OR (lambrolizumab[Title/Abstract])) OR ("atezolizumab" [Supplementary Concept])) OR (anti-PDL1[Title/Abstract])) OR (MPDL3280A[Title/Abstract])) OR (MPDL-3280A[Title/Abstract])) OR (Tecentriq[Title/Abstract])) OR (RG7446[Title/Abstract])) OR (RG-7446[Title/Abstract])) OR ("durvalumab" [Supplementary Concept])) OR (MEDI4736[Title/Abstract])) OR (MEDI-4736[Title/Abstract])) OR (Imfinzi[Title/Abstract])) OR ("Nivolumab"[Mesh])) OR (Opdivo[Title/Abstract])) OR (ONO-4538[Title/Abstract])) OR (ONO 4538[Title/Abstract])) OR (ONO4538[Title/Abstract])) OR (MDX-1106[Title/Abstract])) OR (MDX 1106[Title/Abstract])) OR (MDX1106[Title/Abstract])) OR (BMS-936558[Title/Abstract])) OR (BMS 936558[Title/Abstract])) OR (BMS936558[Title/Abstract])) OR (anti-PD1[Title/Abstract])) OR (PD-1[Title/Abstract])) OR (Programmed Death 1[Title/Abstract])) OR (Programmed Cell Death 1 Receptor[Title/Abstract])) OR (PD1[Title/Abstract])) OR (Programmed Death-Ligand 1[Title/Abstract])) OR (PD-L1[Title/Abstract])) OR (programmed cell death 1 ligand 1 protein[Title/Abstract])) OR (PD L1[Title/Abstract])) OR (PDL1[Title/Abstract])))) OR ("Ipilimumab"[Mesh])) OR (Anti-CTLA-4 MAb Ipilimumab[Title/Abstract])) OR (Anti CTLA 4 MAb Ipilimumab[Title/Abstract])) OR (Ipilimumab, Anti-CTLA-4 MAb[Title/Abstract])) OR (Yervoy[Title/Abstract])) OR (MDX 010[Title/Abstract])) OR (MDX010[Title/Abstract])) OR (MDX-010[Title/Abstract])) OR (MDX-CTLA-4[Title/Abstract])) OR (MDX CTLA 4[Title/Abstract]) AND (((((("Small Cell Lung Carcinoma"[MeSH]) OR (Small Cell Lung Cancer[Title/Abstract])) OR (Oat Cell Lung Cancer[Title/Abstract])) OR (Small Cell Cancer Of The Lung[Title/Abstract])) OR (Carcinoma, Small Cell Lung[Title/Abstract])) OR (Oat Cell Carcinoma of Lung[Title/Abstract]))

EMBASE: 13427 results

(‘pembrolizumab’/exp OR ‘lambrolizumab’:ab,ti OR ‘Keytruda’:ab,ti OR ‘MK-3475’:ab,ti OR ‘atezolizumab’/exp OR ‘anti PDL1’:ab,ti OR ‘MPDL3280A’:ab,ti OR ‘MPDL-3280A’:ab,ti OR ‘Tecentriq’:ab,ti’ OR ‘RG7446’:ab,ti OR ‘RG-7446’:ab,ti OR ‘durvalumab’/exp OR ‘MEDI4736’:ab,ti OR ‘MEDI-4736’:ab,ti OR ‘Imfinzi’:ab,ti OR ‘nivolumab’/exp OR ‘Opdivo’:ab,ti OR ‘ONO-4538’:ab,ti OR ‘ONO 4538’:ab,ti OR ‘ONO4538’:ab,ti OR ‘MDX-1106’:ab,ti OR ‘MDX 1106’:ab,ti OR ‘MDX1106’:ab,ti OR ‘BMS-936558’:ab,ti OR ‘BMS 936558’:ab,ti OR ‘BMS936558’:ab,ti OR ‘anti-PD1’:ab,ti OR ‘PD-1’:ab,ti OR ‘Programmed Death 1’:ab,ti OR ‘Programmed Cell Death 1 Receptor’:ab,ti OR ‘PD1’:ab,ti OR ‘Programmed Death-Ligand 1’:ab,ti OR ‘PD-L1’:ab,ti OR ‘programmed cell death 1 ligand 1 protein’:ab,ti OR ‘PDL1’:ab,ti OR ‘PD L1’:ab,ti OR ‘ipilimumab’/exp OR ‘Anti-CTLA-4 MAb Ipilimumab’:ab,ti OR ‘Anti CTLA 4 MAb Ipilimumab’:ab,ti OR ‘Ipilimumab, Anti-CTLA-4 Mab’:ab,ti OR ‘Yervoy’:ab,ti OR ‘MDX 010’:ab,ti OR ‘MDX010’:ab,ti OR ‘MDX-010’:ab,ti OR ‘MDX-CTLA-4’:ab,ti OR ‘MDX CTLA 4’:ab,ti) AND (‘small cell lung cancer’/exp OR ‘Oat Cell Lung Cancer’:ab,ti OR ‘Small Cell Cancer Of The Lung’:ab,ti OR ‘Carcinoma, Small Cell Lung’:ab,ti OR ‘Oat Cell Carcinoma of Lung’:ab,ti)

Cochrane database: 1716 results

#1 MeSH descriptor: [Nivolumab] explode all trees

#2 MeSH descriptor: [Ipilimumab] explode all trees

#3 MeSH descriptor: [Small Cell Lung Carcinoma] explode all trees

#4 “pembrolizumab” or “SCH-900475” or “Keytruda” or “MK-3475” or “lambrolizumab” or “atezolizumab” or “anti-PDL1” or “MPDL3280A” or “MPDL-3280A” or “Tecentriq” or “RG7446” or “RG-7446” or “durvalumab” or “MEDI4736” or “MEDI-4736” or “Imfinzi” or “Opdivo” or “ONO-4538” or “ONO 4538” or “ONO4538” or “MDX-1106” or “MDX 1106” or “MDX1106” or “BMS-936558” or “BMS 936558” or “BMS936558” or “anti-PD1” or “PD-1” or “Programmed Death 1” or “Programmed Cell Death 1 Receptor” or “PD1” or “Programmed Death-Ligand 1” or “PD-L1” or “programmed cell death 1 ligand 1 protein” or “PDL1” or “PD L1” or “Anti-CTLA-4 MAb Ipilimumab” or “Anti CTLA 4 MAb Ipilimumab” or “Ipilimumab, Anti-CTLA-4 Mab” or “Yervoy” or “MDX 010” or “MDX010” or “MDX-010” or “MDX-CTLA-4” or “MDX CTLA 4”

#5 “Small Cell Lung Cancer” or “Oat Cell Lung Cancer” or “Small Cell Cancer Of The Lung” or “Carcinoma, Small Cell Lung” or “Oat Cell Carcinoma of Lung”

#6 (#1 OR #2 OR #4) AND (#3 OR #5)
